# Dynamic binding of the bacterial chaperone Trigger factor to translating ribosomes in *Escherichia coli*

**DOI:** 10.1073/pnas.2409536121

**Published:** 2024-12-31

**Authors:** Tora Hävermark, Mikhail Metelev, Erik Lundin, Ivan L. Volkov, Magnus Johansson

**Affiliations:** ^a^Department of Cell & Molecular Biology, Uppsala University, Uppsala SE-75124, Sweden

**Keywords:** co-translational processing, protein folding, single-particle tracking, super-resolution microscopy

## Abstract

The bacterial chaperone Trigger factor (TF) binds to ribosome-nascent chain complexes (RNCs) and cotranslationally aids the folding of proteins in bacteria. Decades of studies have given a broad, but often conflicting, description of the substrate specificity of TF, its RNC-binding dynamics, and competition with other RNC-binding factors, such as the Signal Recognition Particle (SRP). Previous RNC-binding kinetics experiments were commonly conducted on stalled RNCs in reconstituted systems, and consequently, may not be representative of the interaction of TF with ribosomes translating mRNA in the cytoplasm of the cell. Here, we used single-particle tracking (SPT) to measure TF binding to actively translating ribosomes inside living *Escherichia coli*. In cells, TF displays distinct binding modes—longer (ca 1 s) and shorter (ca 50 ms) RNC bindings. Consequently, we conclude that TF, on average, stays bound to the RNC for only a fraction of the translation cycle. Further, binding events are interrupted only by transient excursions to a freely diffusing state (ca 40 ms), suggesting a highly dynamic binding and unbinding cycle of TF in vivo. We also show that TF competes with SRP for RNC binding, and in doing so, tunes the binding selectivity of SRP.

## Significance

Translation of the genetic code into proteins by ribosomes is essential for life. Due to the risk of protein misfolding, ribosomes require assistance from cotranslational processing factors, but how these factors find their target ribosomes and how they compete for binding is poorly understood. Here, we measured ribosome binding of the cotranslational chaperone Trigger factor (TF) inside living cells. Thanks to our high spatiotemporal resolution tracking of TF, we were able to distinguish different ribosome-binding kinetics of TF in the cellular environment, and found that, on average, TF stays bound to the ribosome for only a fraction of the translation cycle. Our results further suggest that TF binding tunes ribosome binding of the targeting factor Signal Recognition Particle (SRP).

The nascent polypeptide, emerging at the tunnel exit of the ribosome, is the potential substrate for a large number of targeting factors, modifying enzymes, and chaperones. Depending on the final destination and folding pathway of the mature protein, one or several of these will bind to the ribosome-nascent chain complex (RNC) (1). Trigger factor (TF) is an ATP-independent chaperone that binds to RNCs and cotranslationally aids de novo folding of the nascent chain. Once the polypeptide is released from the ribosome, it may stay associated with TF (2, 3), get handed over to other cytosolic chaperones (4, 5), or fold independently (6). TF has a large substrate pool including cytosolic and secretory proteins, with a preference for sequences of positively charged amino acids flanked by aromatic residues (7), and is particularly enriched on ribosomes translating outer membrane proteins (8). In contrast, TF is underrepresented on ribosomes translating inner membrane proteins (8), which instead are targeted by SRP to the SecYEG translocon for cotranslational insertion into the inner membrane (9). SRP and TF share a docking site on the ribosome close to the polypeptide exit tunnel (10–12). Whether they compete for binding or can bind simultaneously to the same ribosome is a long-standing controversy, as there are studies supporting both cases (13–18).

TF binds to the ribosome via its N-terminal signature GFRxGxxP motif (12). The C-terminal domain forms the center of the structure with two protruding arms and facilitates most interactions with the nascent chain (10, 19–21). The middle domain has peptidyl-prolyl isomerase activity as shown by in vitro experiments (18, 20, 21). The affinity of TF to RNCs is substrate-dependent and recognition of a target nascent chain increases the affinity up to 30-fold compared to a nontarget RNC (13, 22, 23). However, the times reported for target binding vary greatly, from 1 s to 50 s (13, 22–24). A dwell time of 1 s or 50 s on the RNC would have vastly different implications for the in vivo function of TF in the aspect of cotranslational processing, given an average translation rate of 15 to 20 amino acids per second. The first scenario would allow for sequential RNC binding by additional cotranslational processing factors, whereas the latter would require concurrent binding. Again, since there is a lack of consensus regarding the RNC binding competition between the cotranslational processing factors, it is difficult to extrapolate these kinetic parameters to the cellular context. Further, conventional RNC binding kinetics experiments are typically performed in reconstituted systems with ribosomes stalled on the mRNAs, and consequently may not reflect the kinetics of TF interacting with a growing nascent chain. TF-RNC binding kinetics in live cells was studied already in 2016 using in vivo single-particle tracking (SPT). There, TF-RNC binding time in vivo was estimated to be on average ca 0.2 s, but the authors acknowledged that this time represents an average of binding to target RNCs as well as unspecific binding to nontargets (25).

In this work, we have performed SPT of TF in live *Escherichia coli* using an improved fluorescence labeling strategy and with higher temporal resolution, enabling the distinction of different RNC binding modes of TF in vivo. Our labeling approach yields long enough trajectories to capture the transient nature of TF binding and unbinding RNCs within the same trajectory. With this experimental methodology, we establish that the interaction between TF and RNCs in vivo is highly dynamic with mostly rapid binding and unbinding. Moreover, we show that TF competes with SRP for RNC binding, and in doing so, TF tunes the binding selectivity of SRP.

## Results

### SPT Captures RNC-Bound and Free TF.

**Fig. 1.**
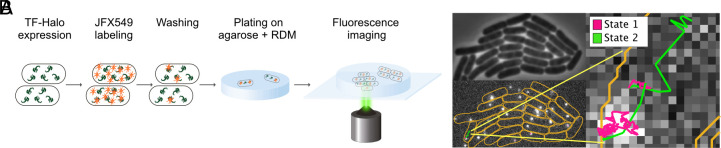
Experimental setup. (*A*) Cells expressing TF-Halo were incubated with JFX549 and spread on agarose pads containing rich defined medium (RDM) with subsequent incubation at 37 °C, allowing formation of small colonies from single cells. Colonies were imaged in phase contrast followed by fluorescence time-lapse movies. (*B*) Trajectories of single TF-Halo particles were assigned to individual cells (*Bottom left*), segmented based on phase contrast images (*Top left*), and fitted to HMM-based models describing discrete diffusion states (*Right*). The trajectory shown is fitted to a 2-state diffusion model, with steps assigned to state 1 (slow diffusion) and state 2 (fast diffusion) color-coded in magenta and green, respectively. Orange lines are cell outlines. To clearly illustrate the diffusional differences in one trajectory, the displayed trajectory is longer (155 steps, 775 ms) than the average trajectory (29 frames).

For SPT of TF in live *E. coli*, we first inserted the gene encoding HaloTag downstream of the gene encoding TF on the chromosome, resulting in a C-terminal fusion (TF-Halo). HaloTag covalently binds chloroalkane ligands (26) such as the chloroalkane-activated organic fluorescent Janelia-Fluor (JF) dyes (27). We have previously confirmed that the TF-Halo fusion is functional in vivo [Supplementary Note 10B in reference (28)]. We applied our SPT method previously used for HaloTag-labeled ribosomes in live *E. coli* (29). In short, cells expressing TF-Halo were grown to exponential phase and then incubated with JFX549. Due to the abundance of TF-Halo, we optimized the concentration of JFX549 to ensure that only a subset of TF-Halo molecules was fluorescent, allowing tracking of a single TF-Halo molecule per cell. Cells were sparsely spread onto an agarose pad prepared with growth media on a microscopy slide (Fig. 1*A*). The sample was incubated at 37 °C for at least 1.5 h before imaging, allowing single cells to divide and form small colonies, thus capturing TF-Halo dynamics in growing cells with active translation.

We performed stroboscopic fluorescence imaging of colonies using a camera exposure time of 5 ms with 3 ms laser illumination per frame. The fluorescence time-lapse movies were analyzed with our pipeline previously used for SPT of tRNA (30), ribosomal subunits (29), and SRP (31). In short, cell outlines were identified based on phase contrast images and a dot detection algorithm identified fluorescing TF-Halo dots in each frame of the movie. Diffusion trajectories of single fluorescent dots were assigned to individual cells, describing the diffusional behavior of the particle over time (Fig. 1*B*) with a mean trajectory length of 29 frames (*SI Appendix*, Fig. S1).

We observed a distinct diffusional pattern, where the particles were mostly close to immobile and only briefly transitioned into a faster diffusional state (Movie S1). Based on our expectations of TF function, we hypothesized that the slow diffusion corresponds to RNC binding and that the faster diffusion is free TF-Halo. To quantify these diffusion differences, we applied a Hidden Markov Modeling (HMM) approach in which all trajectories are fitted to a predefined number of discrete diffusion states based on the trajectory step lengths, with state diffusion coefficients and transition frequencies between these states as fitting parameters (29–31). Each diffusion state, hence, is described by an average diffusion coefficient (D), the steady-state fraction of particles in said state (occupancy), and the average time spent in that state (dwell time).

**Fig. 2.**
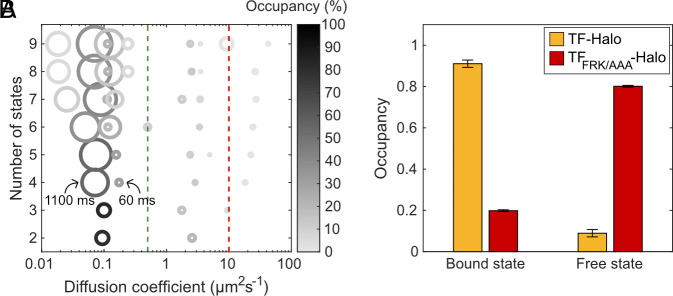
SPT distinguishes free and RNC-bound TF-Halo. (*A*) HMM fitting of trajectories to different model sizes (2–9). Each model is resolved along the y-axis with discrete diffusion states represented by circles resolved along the x-axis based on diffusion rate. Circles are color-coded according to state occupancy and the area is proportional to the state dwell time. As reference points, two dwell times in the 4-state model are explicitly indicated. Green and red lines mark diffusion thresholds of 0.5 and 10 µm^2^s^−1^, respectively, separating the three clusters of diffusion states (RNC binding, free TF-Halo, and tracking artifacts). Data show chromosomally expressed TF-Halo, n = 99,352 trajectory steps cumulated from 3 independent experiments. Full HMM output is found in Dataset S1, Tab 4. (*B*) Occupancy in 2-state models of TF-Halo and TF_FRK/AAA_-Halo, expressed from plasmids in a TF knockout strain. The 2-state models are weighted averages of models with 4 to 9 states coarse-grained into two states using a threshold of 0.5 µm^2^s^−1^ to separate RNC binding from free TF-Halo. n = 83,546 and 75,585 trajectory steps from 3 and 5 independent experiments for TF-Halo and TF_FRK/AAA_-Halo, respectively. Error bars represent the weighted SD calculated from coarse-grained models of 4 to 9 states. Full output of coarse-grained models is shown in Dataset S1, Tab 5, and output from all model sizes is shown in Dataset S1, Tabs 6 and 7.

The HMM algorithm does not provide which model size best fits the data. Unfortunately, statistical criteria for choosing the best model size, such as Akaike’s information criterion (AIC) or Bayesian information criterion (BIC), are also not very helpful in the assessment of biological diffusion trajectories. While AIC and BIC may perform well on simplified model diffusion systems or simulated data with well-defined discrete diffusion rates, a molecule’s motion in a cell is in many cases rather heterogenous and best described by a set of diffusion distributions rather than discrete diffusion states. Hence, the more data we have available, increasingly complex models will fit the data better than lower-complexity models, when evaluated with AIC (*SI Appendix,* Fig. S2) (29, 32). Instead, in the absence of a reliable statistical criterion, we find that an empirical assessment of different model sizes, combined with biological control experiments, is the best way to find the model size that robustly describes the underlying data and has biological relevance. We thus fitted the data to models with increasing sizes, ranging from 2 to 9 diffusion states (Fig. 2*A* and Dataset S1, Tabs 1–4) (29), and found that three clusters of states appeared: one cluster of ribosome-like diffusion states (29, 33) in the range 0.02 to 0.2 µm^2^s^−1^, a second cluster with tenfold faster diffusion ranging from 2 to 5 µm^2^s^−1^, potentially corresponding to freely diffusing TF-Halo, and a third cluster containing low-occupancy states (<1%) with diffusion rates too high to be physiologically relevant (>10 µm^2^s^−1^). We assign the third cluster to artifacts of the trajectory building, also seen in the analysis of simulated microscopy data (30), appearing, for example, due to low-frequency erroneous linking of two different dots in one trajectory (*SI Appendix*, Fig. S3).

To specifically investigate RNC binding events, and to remove any model dependence, we coarse-grained the larger HMM-fitted models into 2 states using a threshold at 0.5 µm^2^s^−1^ (*SI Appendix*, Note S1) (29–31, 33), with state 1 corresponding to RNC-bound TF-Halo and state 2 to free TF-Halo. The occupancy and average dwell time, calculated as weighted averages from coarse-grained models 4 to 9, were 85% and 180 ms for the bound state, and 15% and 28 ms for the free state, respectively. To verify that the slow diffusion state captures RNC binding, we tracked a TF mutant in which residues 44 to 47 (FRK) were exchanged to AAA, thus compromising RNC binding (12, 25). Indeed, TF_FRK/AAA_-Halo displayed a slow-state occupancy of only 20%, confirming that slow diffusion corresponds to RNC binding by TF (Fig. 2*B* and *SI Appendix,* Fig. S4 and Movie S2).

### RNC Binding Kinetics Depend on the Cellular Level of TF-Halo.

**Fig. 3.**
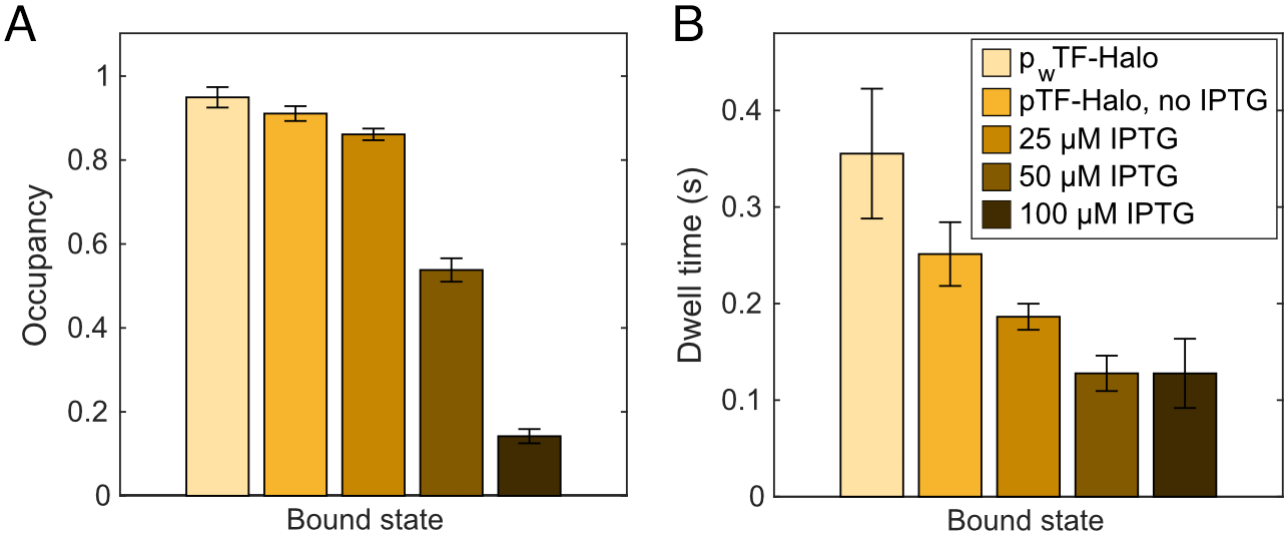
Increasing TF-Halo levels reduce the occupancy and average dwell time of RNC binding. Occupancy (*A*) and dwell time (*B*) in the RNC-bound state in 2-state models. TF-Halo was expressed from plasmids in a TF knockout strain, either with weak constitutive (p_w_TF-Halo) or IPTG-inducible expression (pTF-Halo with 0, 25, 50, and 100 µM IPTG). The 2-state models are weighted averages of models with 4 to 9 states coarse-grained into two states using a threshold of 0.5 µm^2^s^−1^. n = 110,271; 83,546; 107,952; 86,308; 62,507 steps from 3, 3, 3, 3, and 4 independent experiments for each condition as listed in the figure legend. Error bars represent the weighted SD calculated from coarse-grained models of 4 to 9 states. Full output from coarse-grained models is shown in Dataset S1, Tab 5, and output from all model sizes in *SI Appendix*, Tables S7–S11.

The RNC-bound dwell time in the coarse-grained 2-state model, 180 ms, likely represents an average of a broad distribution of high- and weak-affinity binding to different RNC species, as expected from in vitro binding assays (13, 22–24) and in line with previous in vivo TF SPT results (25). To investigate whether the average RNC-bound time could be perturbed, we titrated the TF-Halo levels in a TF knockout strain (Fig. 3 and Dataset S1, Tabs 5 and 7–11). With gradual increase of TF-Halo, the RNC-bound dwell time decreases significantly, with proportionally less effect on the RNC-bound occupancy, supporting that the average dwell time in this 2-state model comprises a broad distribution of dwell times. The decrease in the average bound time suggests that shorter bindings are enriched when the number of TF-Halo molecules is increased. Further, at higher expression levels, the system becomes saturated with respect to vacant RNC binding sites, as reflected by a drastic decrease also in the RNC-bound occupancy.

**Fig. 4.**
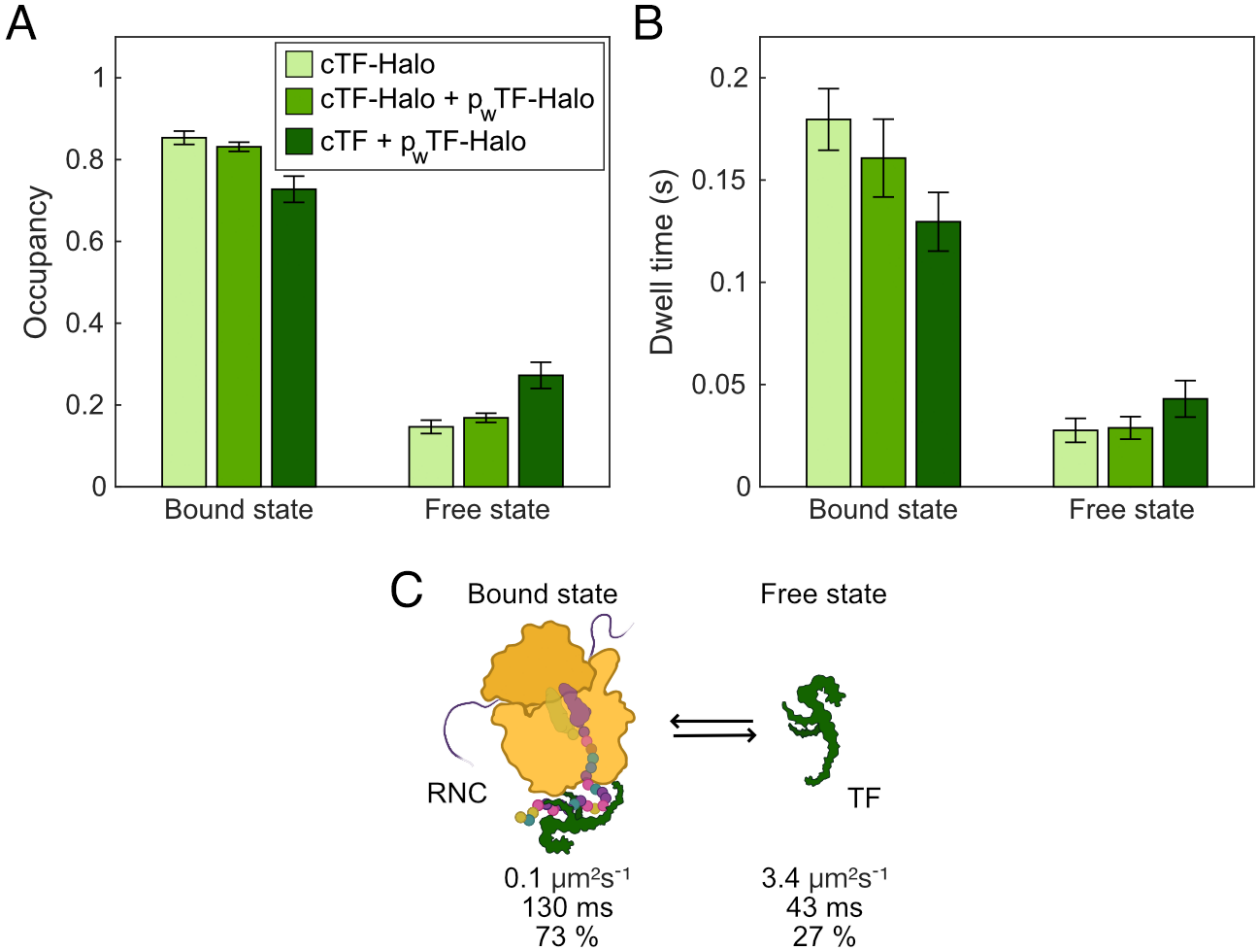
2-state coarse-grained models of TF-Halo in different background strains. Occupancy (*A*) and dwell time (*B*) in 2-state models from SPT of TF-Halo in the chromosomally tagged strain (cTF-Halo), the chromosomally tagged strain with additional weak plasmid expression (cTF-Halo + p_w_TF-Halo) and in a wt strain with additional weak plasmid expression (cTF + p_w_TF-Halo). (*C*) 2-state model based on cTF + p_w_TF-Halo. The 2-state models are weighted averages of models with 4 to 9 states coarse-grained into 2 states using a threshold of 0.5 µm^2^s^−1^. n = 99,352; 92,876; 96,321 trajectory steps cumulated from 3, 3, and 4 independent experiments for cTF-Halo, cTF-Halo + p_w_TF-Halo, and cTF + p_w_TF-Halo, respectively. Error bars represent the weighted SD calculated from coarse-grained models of 4 to 9 states. Full output from coarse-grained models is shown in Dataset S1, Tab 5, and output from all model sizes is shown in Dataset S1, Tabs 4, 12, and 13.

These titration experiments highlight that the kinetic parameters in our coarse-grained 2-state models depend on the number of TF molecules competing for RNC binding. In order to investigate whether the stoichiometry between RNCs and TF-Halo in the chromosomally tagged strain is representative of wt cells, we also performed SPT of TF-Halo expressed in low copy number (*SI Appendix,* Fig. S5) from a plasmid in a wild-type (wt) TF background, as well as in the chromosomal TF-Halo background. We find that the RNC-bound state occupancy of TF-Halo is slightly lower when competing with wt TF (73%) than when competing with chromosomally expressed additional TF-Halo (83%, Fig. 4*A* and Dataset S1, Tabs 12 and 13). This suggests that the cellular level of TF-Halo is lower than that of wt TF when they are expressed from the same position on the chromosome, or, alternatively, that TF-Halo has slightly reduced binding capacity to RNCs compared to the wt TF. However, since TF-Halo is functional (28), and since the overall RNC binding dynamics of TF-Halo is highly similar in all three scenarios (Fig. 4*B*, average bound-state dwell times 130 to 180 ms, and free-state dwell time 28 to 43 ms), we believe that our measurements represent the binding kinetics of TF to RNCs well. The parameters measured from low-level TF-Halo in the wt TF background (Fig. 4*C*), though, are likely the most accurate.

### TF Displays Distinct RNC Binding Modes In Vivo.

**Fig. 5.**
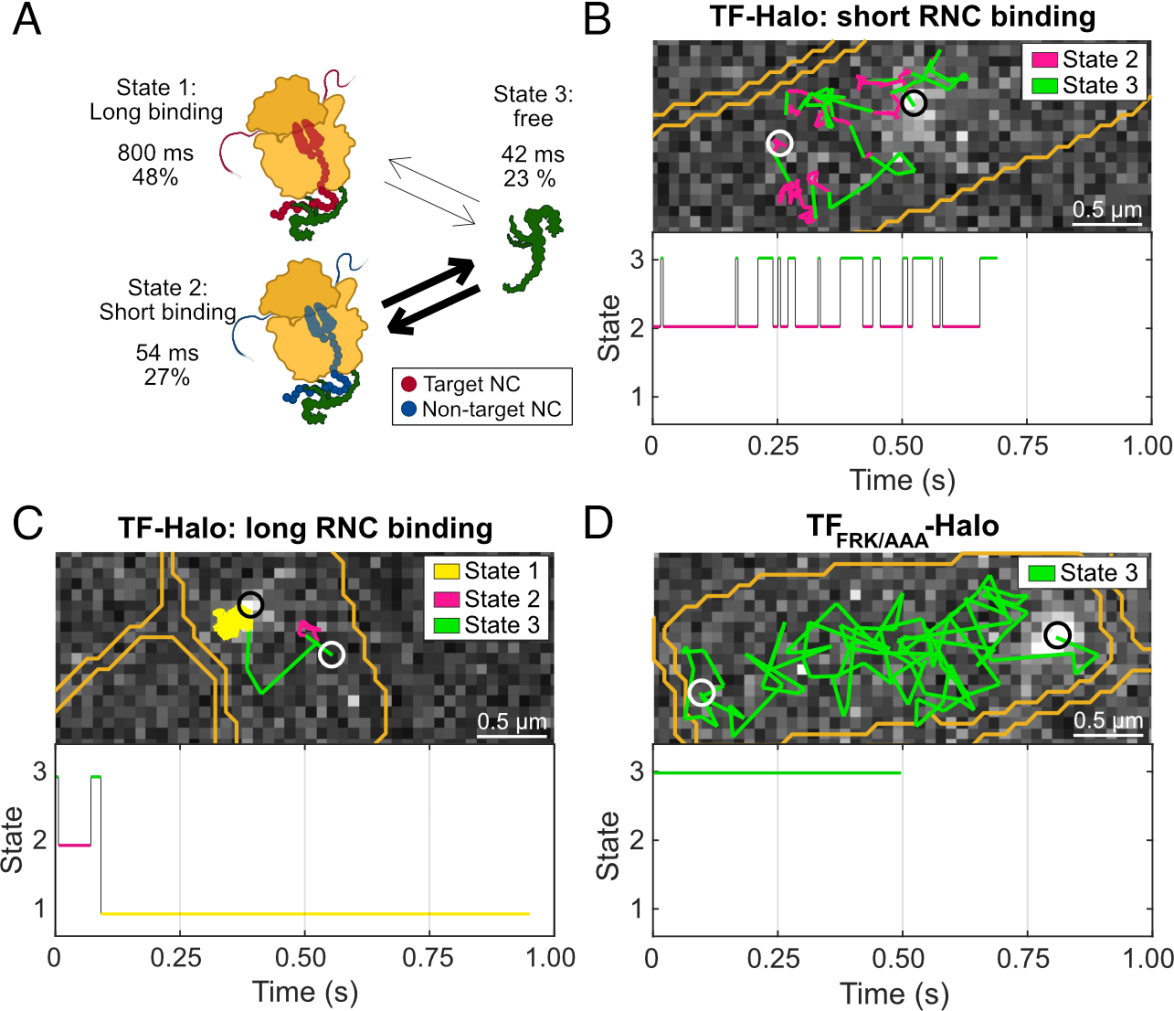
Higher-complexity models resolve two distinct RNC binding modes. (*A*) Biological interpretation of 4-state model. The thickness of the arrows is proportional to the relative fluxes of particles between states. State 4, with D = 12 µm^2^s^−1^ and 2% occupancy, is assigned to tracking artifacts and thus not included in the biological interpretation. The complete model with state 4 included is shown in *SI Appendix*, Fig. S9 and Dataset S1, Tab 13. The model is based on SPT of low levels of TF-Halo in a wt TF background (cTF + p_w_TF-Halo), n = 96,321 trajectory steps cumulated from 4 independent experiments. A similar 4-state model was obtained from chromosomally expressed TF-Halo (Dataset S1, Tab 4). (*B*) Example of TF-Halo trajectory displaying fast transitions between free diffusion and short-lived RNC binding. c Example of a TF-Halo trajectory displaying longer RNC binding. d Example trajectory of TF_FRK/AAA_-Halo. Trajectories in (*B*–*D*) are HMM-fitted to 4-state models. The top panels display trajectories in space and the bottom panels show the state transitions in each trajectory over time. Steps are color-coded according to state assignments, with states 1, 2, and 3 corresponding to long RNC binding, short RNC binding, and free TF-Halo, respectively. White and black circles indicate start and end points of a trajectory, respectively. Cell outlines are shown in orange. The displayed trajectories were chosen to clearly illustrate the different binding modes within one trajectory. Hence, they are longer than the average trajectory (*SI Appendix*, Fig. S1). More example trajectories are shown in *SI Appendix*, Fig. S8. Further, statistics on the number of trajectories and transitions included in the analysis on a per-state basis are presented in *SI Appendix*, Tables S8–S13.

From the coarse-grained 2-state analysis of TF-Halo diffusion, we established that TF is RNC-bound 73% of the time with an average dwell time of 130 ms (Fig. 4*C*, low TF-Halo expression in a wt background), which we assume is a global average of a broad distribution of high- and weak-affinity binding to different RNC species. However, when inspecting more complex models (≥4 states), we robustly identified one RNC-bound state with significant occupancy (around 30%) and approximately 10 to 20-fold shorter average dwell time than the other RNC-bound states (see detailed description in *SI Appendix*, Fig. S6 for chromosomally expressed TF-Halo, and *SI Appendix*, Fig. S7 for low-level TF-Halo in a wt background). We speculate that these long- and short-lived RNC-bound states are separated in the HMM analysis not due to differences in diffusion rates, but rather, different underlying binding kinetics, as expected from previous kinetics studies on TF to different RNC species. Since the 4-state model is the simplest one where the distinct RNC-bound states appear, both for chromosomally expressed TF-Halo (Fig. 2*A* and Dataset S1, Tab 4) and for low TF-Halo expression in a wt background (*SI Appendix,* Fig. S7 and Dataset S1, Tab 13), we investigated this model further. For low TF-Halo expression in a wt background, state 4 is fast (D_4_ = 12 µm^2^s^−1^) with 2% occupancy, corresponding to the aforementioned tracking artifacts, and is, hence, disregarded from the biological interpretation. We tentatively assign free TF-Halo to state 3 (D_3_ = 3 µm^2^s^−1^), shorter RNC binding to state 2 (D_2_ = 0.2 µm^2^s^−1^), and longer RNC binding to state 1 (D_1_ = 0.1 µm^2^s^−1^). According to this model, 48% of all TF-Halo molecules display long binding at steady state with an average dwell time of approximately 800 ms (Fig. 5*A* and Dataset S1, Tab 13). The occupancy and dwell time in the short-lived RNC-bound state is 27% and 54 ms, respectively, whereas 23% of TF-Halo is in the free state where it stays for, on average, 42 ms before binding a new RNC. By inspecting the HMM estimated fluxes of molecules between states, we find frequent transitions between states 2 and 3 (*SI Appendix*, Tables S8 and S9, and as exemplified in Fig. 5*B* and *SI Appendix,* Fig. S8) with occasional long-lasting excursions to state 1 (Fig. 5*C*), also apparent by eye in the fluorescence movies (Movies S3 and S4). This pattern indicates that TF-Halo is exerting distinct RNC binding modes; one in the time range of tens of milliseconds, and one in the one-second range. The short-lived bindings are more frequent than the long-lived, with one long binding occurring per 20 short binding events. The same pattern is observed for the chromosomally expressed TF-Halo, with two RNC-bound states of 1100 ms and 59 ms, respectively (*SI Appendix*, Fig. S8 and Tables S10 and S11 and Dataset S1, Tab 4). In contrast, trajectories of TF_FRK/AAA_-Halo, compromised in ribosome binding, show fast diffusion without any such frequent transitions between states 2 and 3 (Fig. 5*D* and *SI Appendix,* Fig. S8 and Tables S12 and S13 and Movie S5), confirming that both states 1 and 2 capture RNC-binding events rather than any other slow-diffusing, ribosome-independent, activity of TF-Halo.

Further, we confirmed that this model is robust with respect to time resolution by reducing the camera exposure time from 5 ms to 2.5 ms. With 2.5 ms exposures, the distinction between longer and shorter binding modes remained (*SI Appendix*, Fig. S10 and Dataset S1, Tab 14). In contrast, by increasing the exposure time to 60 ms [as in the TF SPT in reference (25)], the distinction disappeared (*SI Appendix*, Fig. S10 and Dataset S1, Tab 15), highlighting why a temporal resolution higher than the average short-lived binding time is necessary for distinguishing the different kinetics. As the short-lived bindings (≤60 ms) are the most frequent type of interaction with the RNCs (ca 20 short bindings per 1 long binding event, *SI Appendix*, Fig. S9), they have a significant influence on the average binding time in the 2-state model obtained from 5 ms tracking (ca 0.2 s). At 60 ms tracking, any such short bindings are too fast to be recorded, and thus, by coarse-graining the 60 ms models into 2 states, we obtain a robust and model-independent estimate of the longer bindings alone (see details in *SI Appendix*, Fig S11). With 60 ms per frame, the average RNC binding in the weighted mean coarse-grained 2-state model is 1.8 s (Dataset S1, Tab 5; for model size robustness, see *SI Appendix,* Fig. S11*C*), i.e., approximately 60% longer than the long-lived RNC-state in the 4-state model estimated for the same expression system at 5 ms (Fig. 5*A*). In our previous analysis of ribosome subunit tracking (29), 60 ms exposures and an identical coarse-graining analysis approach yielded dwell times between 20 to 50 s for translating ribosomes. Hence, these particular microscopy settings and analyses allow the detection of binding events much longer than a few seconds. Since an average translation cycle in *E. coli* at these growth conditions is around 15 s (29), our results suggest that TF, on average, does not linger on the RNC throughout the translation cycle, but rather, displays a more dynamic binding and unbinding. We cannot rule out, however, that longer RNC bindings exist [as suggested by in vitro data (2, 22, 23)], but then, given our data, these must be rare in vivo, such that they do not significantly impact the average RNC-bound time.

**Fig. 6.**
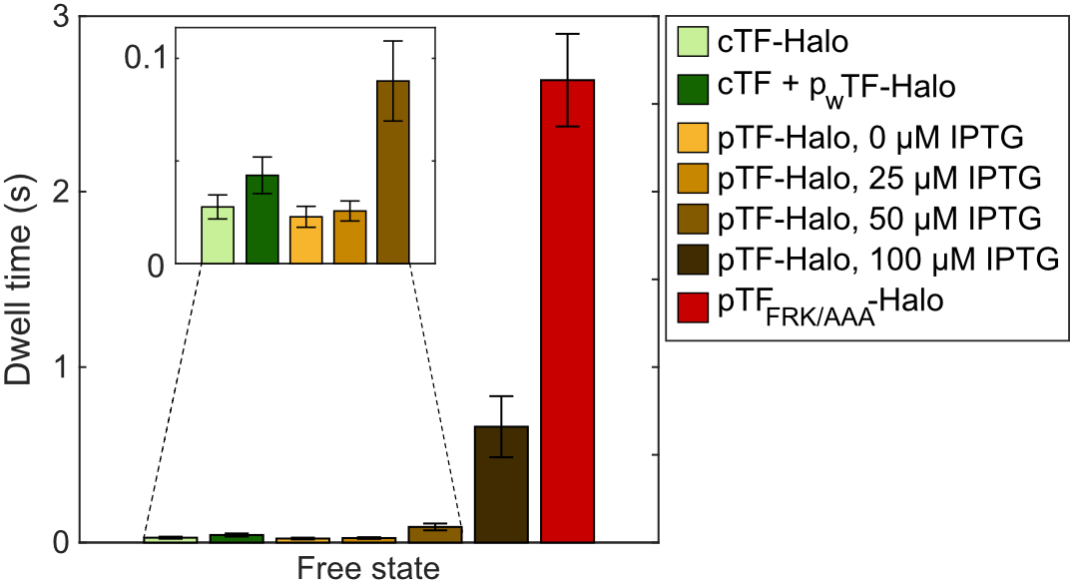
Free state dwell times of TF-Halo under different experimental conditions. Free state dwell times in 2-state models obtained from weighted averages of coarse-grained models (4 to 9) with a threshold of 0.5 µm^2^s^−1^. Conditions with the prefix “c” encode chromosomal expression of TF or TF-Halo, “p_w_” encodes low constitutive expression of TF-Halo from a plasmid and “p” is IPTG-inducible plasmid expression in a TF knockout strain. n = 99,352; 96,321; 83,546; 107,592; 86,308; 62,507; and 75,585 trajectory steps from 3, 4, 3, 3, 3, 4, and 5 independent experiments for each condition as listed in the figure legend. All coarse-grained models are shown in Dataset S1, Tab 5, and full HMM models in Dataset S1, Tabs 4, 6–10, and 13. Error bars represent weighted SD calculated from the included model sizes (4 to 9).

When comparing the kinetic models of TF-Halo and the FRK/AAA mutant, we noticed that by removing the RNC binding ability, and consequently, the fast transitions between free TF-Halo and RNC binding, the free state dwell time in the 2-state models increased by 100-fold, from tens of milliseconds to seconds (Fig. 6). Thus, the free state dwell time is an indicator of whether or not TF is able to bind RNCs. With this observation in mind, we revisited the TF-Halo titration experiments (Fig. 3), where we concluded that TF-Halo compete for RNC binding at high expression levels. Inducing TF-Halo expression with 25 µM Isopropyl β-D-1-thiogalactopyranoside (IPTG) did not affect the free state dwell time, indicating that there are more vacant RNC binding sites than TF-Halo molecules in the cells (Fig. 6). When inducing TF-Halo expression with 50 and 100 µM IPTG, the free-state dwell time increased to 89 ± 20 and 660 ± 170 ms, respectively, confirming our previous conclusion that at such high expression levels, the system is saturated with respect to RNC binding, and TF is diffusing freely for a longer time before finding a vacant RNC to bind to.

### A Nascent Polypeptide Chain Is Needed for TF Binding to Ribosomes.

**Fig. 7.**
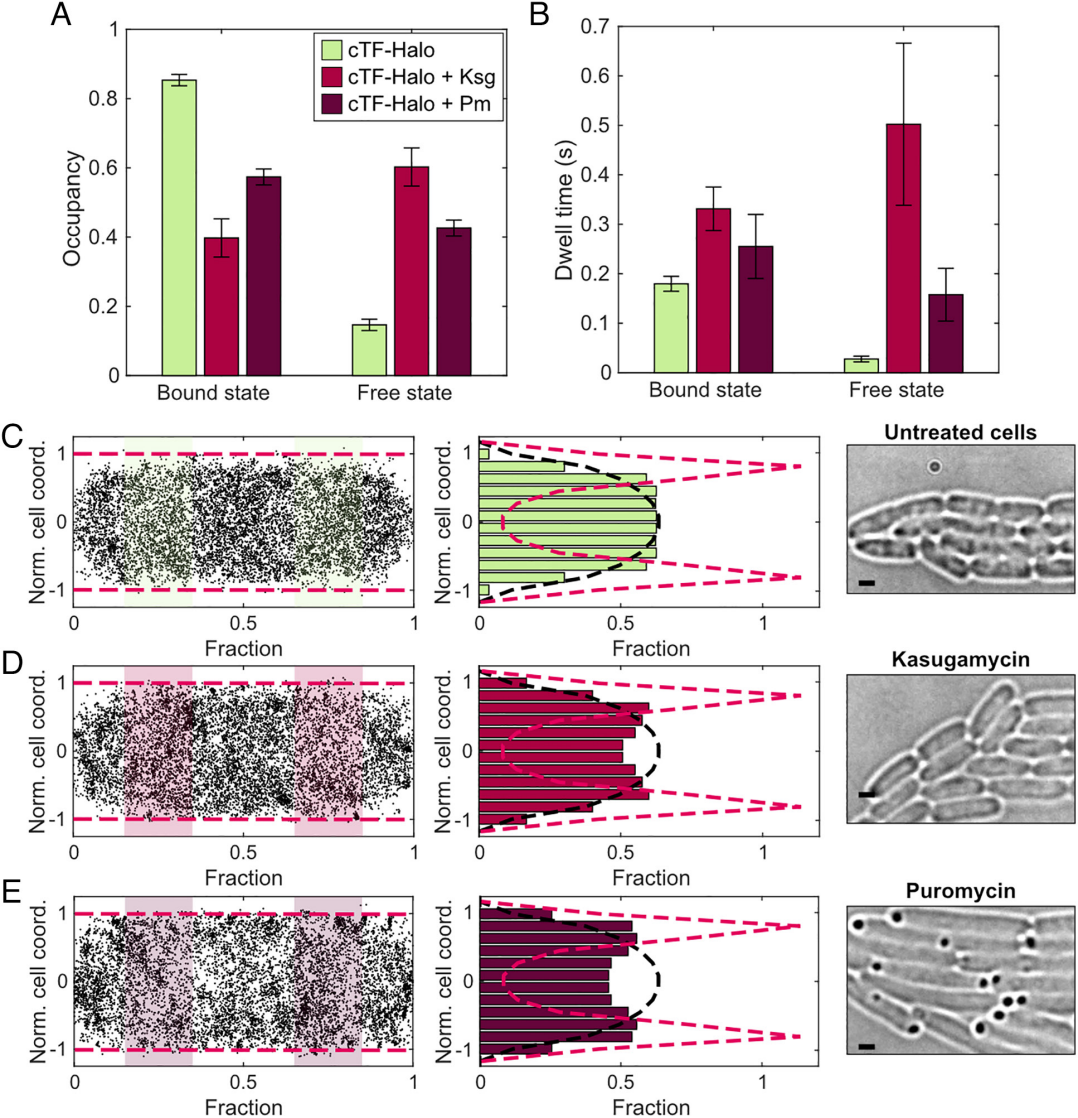
TF-Halo in cells treated with kasugamycin and puromycin. Occupancy (*A*) and dwell time (*B*) in 2-state models from SPT of TF-Halo in the chromosomally tagged strain (cTF-Halo), with 2 mg/ml Ksg or 0.2 mg/ml Pm added to the sample. The 2-state models are weighted averages of models with 4 to 9 states coarse-grained into 2 states using a threshold of 0.5, 0.8, and 1 µm^2^s^−1^ for untreated cells, Ksg and Pm treatment, respectively. The thresholds were determined based on the diffusion rate of L9-Halo in Ksg-treated cells (29) and Pm-treated cells (Dataset S1, Tabs 5 and 18) to separate TF-Halo bound to 50S and 70S ribosomes from free TF-Halo. n = 99,352; 85,146 and 67,028 trajectory steps cumulated from 3, 3, and 5 independent experiments, for untreated, Ksg-treated, and Pm-treated samples, respectively. Error bars represent the weighted SD calculated from coarse-grained models of 4 to 9 states. Full HMM outputs from all model sizes are shown in Dataset S1, Tabs 4, 16, and 19, and 2-state coarse-grained models in *SI Appendix*, Table 5. (*C*–*E*) Spatial distribution of slow-diffusing TF-Halo in untreated cells (*C*), and in cells treated with Ksg (*D*) and Pm (*E*). The left panels show coordinates of detected dots assigned to the slow state (state 1) in a normalized cell geometry. For plotting, 10,000 dots were selected randomly. Magenta dashed lines mark the edges of the normalized cell. The middle panels show the radial distribution (green, light, and dark purple) of all dots in the shaded areas (to exclude the cell poles). The magenta dashed line is the radial profile of a membrane-bound protein derived experimentally from LacY-Halo tracking (*SI Appendix*, Fig. S14) and the black dashed line is a theoretically derived profile of a cytosolic particle. Data are from state 1 of the 9-state models coarse-grained into 2 states, using the same thresholds as in a and b. The right panels show brightfield images of cells in each experimental condition. The dark spots observed in the Pm-treated cells indicate severe protein aggregation. The scale bar corresponds to 1 µm.

Previous in vitro studies have shown that TF displays low-affinity binding to vacant, nontranslating, 70S ribosomes, with a dissociation rate similar as for RNCs displaying nontarget nascent chains (13, 23). To our knowledge, the binding kinetics of TF to free 50S subunits have not been measured in vitro, but stable TF binding to free 50S subunits has been captured for structural analyses (10, 34, 35). Thus, in the previous in vivo TF SPT study (25), it was concluded that interactions of TF with free 50S subunits were included in the diffusion model. However, we have reason to believe that this is a misinterpretation of the data due to a limitation in time resolution, as discussed in detail in *SI Appendix*, Note S2. To investigate the nature of TF-Halo interactions with nontranslating ribosomes, we treated cells with kasugamycin (Ksg), an inhibitor of translation initiation (36). Our previous SPT of ribosomal subunits showed that, under identical Ksg treatment conditions, 80% of the 50S subunits are freely diffusing (29). Upon Ksg treatment, the overall bound-state occupancy of TF decreased from 85 ± 2% to 40 ± 6% (Fig. 7*A* and *SI Appendix*, Fig. S12 and Dataset S1, Tabs 5 and 16). Further, the free-state dwell time increased drastically from 28 ± 6 ms to 500 ± 160 ms (Fig. 7*B*), similar to what was observed for TF-Halo overexpression (Fig. 6, induction with 100 µM IPTG), indicating a reduction in binding sites. If TF binds nontranslating ribosomes equally well as elongating 70S ribosomes, we would not observe such an effect, as the number of possible binding sites, whether they be in 50S or 70S configuration, would be constant. Hence, we conclude that TF-Halo binds to nontranslating 50S subunits less efficiently than to elongating ribosomes. We cannot exclude, however, that there are rapid binding events also to nontranslating ribosomes and free 50S subunits, but then these binding events must be faster than the temporal resolution of our experiments, i.e., less than a few milliseconds.

To corroborate the results from the Ksg treatment, we also tracked TF-Halo and ribosomes in cells treated with puromycin (Pm), an antibiotic mimicking aminoacyl-tRNA, causing premature translation termination and nascent chain release (37, 38). First, we tracked HaloTag-labeled 50S subunits [labeled on ribosomal protein L9 (29)] with and without Pm treatment, to better understand the effect of Pm on the diffusion of ribosomes in vivo. Upon Pm treatment, 33 ± 3% of the 50S subunits are freely diffusing (compared to 11 ± 1% in untreated cells, Dataset S1, Tabs 5, 17, and 18), i.e., a less pronounced effect than for the Ksg treatment [80% 50S (29)]. The same trend was observed for TF-Halo in Pm-treated cells, with a reduction in the bound state occupancy from 85 ± 2% to 57 ± 2% (Fig. 7*A*) and an increase in the free-state dwell time from 28 ± 6 ms to 158 ± 53 ms (Fig. 7*B*). Thus, Pm treatment causes similar but less pronounced effects on the RNC binding of TF-Halo as the Ksg treatment. These experiments provide further support of our interpretation from TF-Halo tracking in the presence of Ksg, i.e., that a nascent peptide is needed for TF binding to ribosomes. Interestingly, in addition, we noticed a pronounced shift in the spatial distribution of slow-diffusing TF-Halo upon Pm treatment toward the membrane (Fig. 7*E*). In contrast, there was no significant effect on the distribution of ribosomes toward the membrane (*SI Appendix*, Note S3), suggesting that this slow-diffusing membrane-proximate activity of TF-Halo is not cotranslational. While a small spatial effect on the slow-diffusional state of TF-Halo is seen also with Ksg (Fig. 7*D*), the shift is not significant compared to untreated cells (*SI Appendix*, Note S3). Kasugamycin acts by inhibiting translation initiation, whereas puromycin causes premature translation termination, and consequently, accumulation of truncated and misfolded proteins (as confirmed by dark spots in brightfield images, Fig. 7*E*). Thus, we propose that the increased membrane dwelling of TF-Halo observed in the presence of Pm is TF-Halo binding to protein aggregates.

### TF-Halo and SRP Compete for RNC Binding In Vivo.

**Fig. 8.**
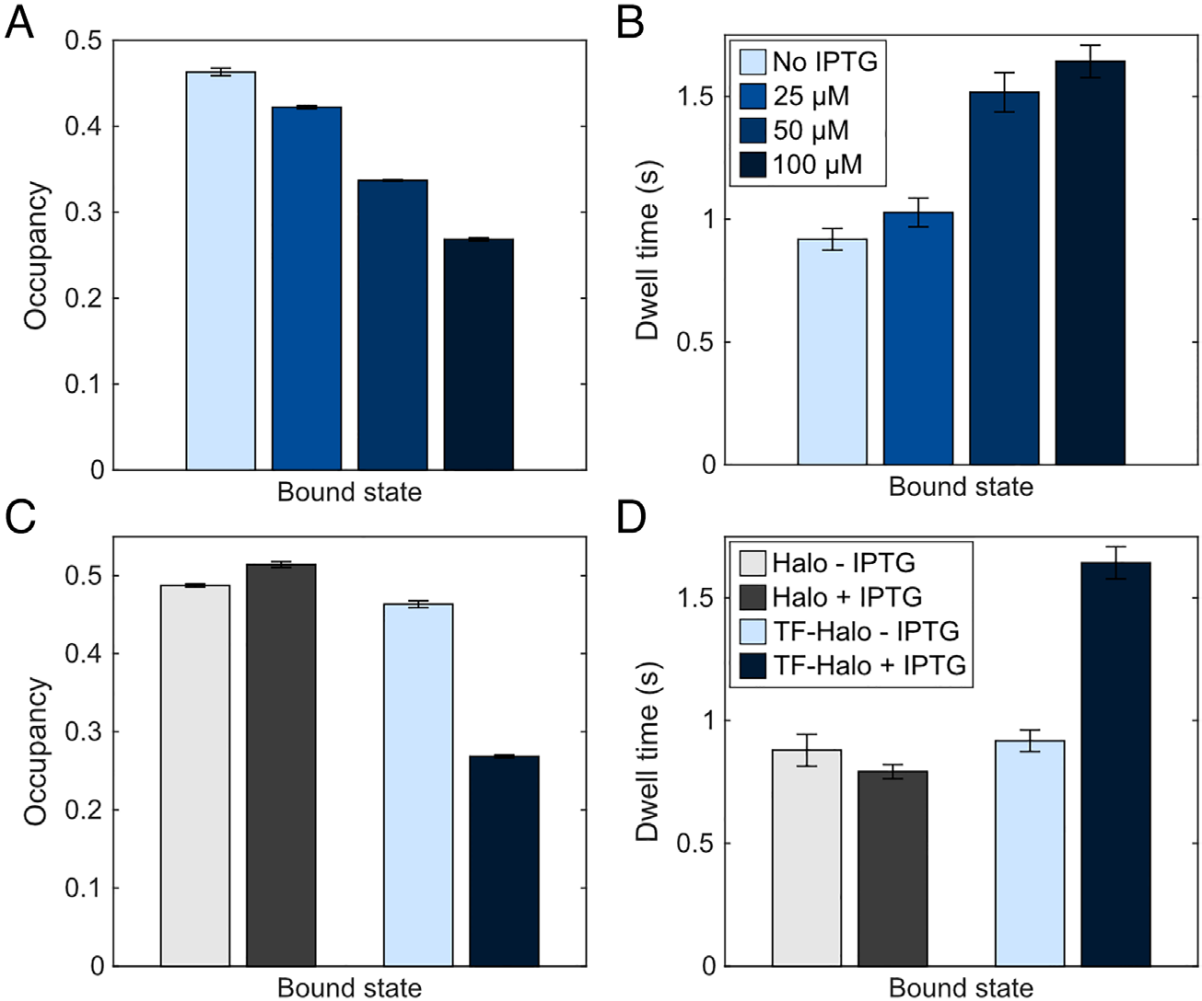
TF-Halo competes with SRP for RNC binding. Occupancy (*A*) and dwell time (*B*) of SRP in the RNC-bound state when inducing expression of (dark) TF-Halo with 0, 25, 50, and 100 µM IPTG from a plasmid in a TF knockout strain. (*C* and *D*) Occupancy and dwell time of SRP in the RNC-bound state with 0 and 100 µM IPTG induction of either HaloTag alone or TF-Halo. Data show 2-state models obtained by weighted averages of models with 4 to 9 states coarse-grained into two states using a threshold of 0.5 µm^2^s^−1^. n = 79,881; 65,506; 54,544; 66,430; 81,499; 106,826 trajectory steps for induction of TF-Halo with 0, 25, 50, and 100 µM and 0 and 100 µM of HaloTag, respectively. Each condition was tested in 3 independent experiments. Error bars represent weighted SD calculated from the included model sizes (4 to 9). HMM output from coarse-grained 2-state models is shown in Dataset S1, Tab 5, and output from all model sizes in Dataset S1, Tabs 20–25.

We next sought to investigate the possible competition for RNC binding between TF and SRP. While this in vivo competition was explored by Yang et al. (25), they studied the effect of increased SRP levels on the RNC binding of TF and confirmed a small but significant decrease in the RNC-bound fraction of TF, suggesting an in vivo binding competition. However, since the cellular levels of TF are much higher than those of SRP [40 to 90 µM TF and approximately 1 µM SRP (39–43)], the inhibitory effect of SRP on TF-RNC binding may be insignificant in a normal cell. Instead, the possible inhibitory effect of TF on the RNC binding kinetics of SRP is a more compelling question. Hence, we applied our protocol for SRP tracking (31) and explored the effect on SRP–RNC binding upon titration of TF-Halo, induced by IPTG, in a TF knockout strain. With increasing TF-Halo concentration, the RNC-bound fraction of SRP decreased, from 46.3 ± 0.4% at the lowest TF-Halo concentration, to 26.8 ± 0.2% at the highest (Fig. 8*A* and Dataset S1, Tabs 20–23). The effect is TF-specific, as no such reduction was seen when HaloTag alone was titrated using the same expression system (Fig. 8*C* and Dataset S1, Tabs 24 and 25). Thus, we conclude that TF and SRP compete for ribosome binding in vivo.

Since TF and SRP have distinct substrate pools, i.e., SRP mainly targets ribosomes translating inner membrane proteins whereas TF targets cytosolic and secretory proteins (8, 9), we first hypothesized that the reduction in RNC binding by SRP is a consequence of increased RNC binding events by TF-Halo, making those RNCs which are translating SRP targets less accessible to SRP. However, we observe that the dwell time of SRP on RNCs gradually increases with elevated TF-Halo levels (Fig. 8*B*), suggesting that at low TF levels, there is a larger fraction of short unproductive SRP–RNC interactions, which consequently reduces the average dwell time. Thus, a more likely explanation to the observed reduction in RNC binding by SRP would be that, at low TF-Halo concentration, SRP also binds TF targets to a substantial extent. Again, the effect is TF specific, since the dwell-time of SRP on ribosomes does not change significantly upon overexpression of HaloTag alone (Fig. 8*D*). Hence, our results imply that in the absence of TF-Halo, SRP displays more unproductive RNC bindings, suggesting that the large number of TF molecules in a normal cell tune the selectivity of SRP. A similar model was proposed from rigorous in vitro experiments by Ariosa et al. (17), and our results confirm that their model is applicable to the cellular context.

## Discussion

TF is an extensively studied chaperone, but there is a lack of consensus regarding its RNC-binding properties, and we have had limited knowledge of its overall activity in the cell. In this work, we report TF binding to translating RNCs in living cells at a high enough temporal resolution to distinguish longer and shorter RNC bindings. Although experiments in reconstituted systems have suggested that differences in binding kinetics occur depending on the nascent chain sequence and length, this work captures them in the native environment, and during ongoing protein synthesis. We find that TF mainly exerts short bindings, on the 50-ms timescale and that longer bindings are, on average, approximately one to two seconds.

The affinity of TF to RNCs will vary, both between the synthesis of different proteins, but also within one protein species, as the nascent chain grows. We thus expect there to be a broad distribution of RNC-bound dwell times in the cell. As such, the long- and short-lived states presented here represent averages of longer and shorter dwell time distributions, respectively. In theory, data fitting to more complex models should distinguish the binding kinetics to different RNC species. Indeed, by adding up to 9 states in our fitting, we obtain several states with slow diffusion, each with different dwell times and occupancies. With higher complexity, the long-lived state is separated into several states with dwell times ranging roughly between 0.1 to 2 s. Although these states may correspond to bindings to different RNC species, we find that the occupancies and transitions between those states are model dependent, and as such, ill-defined. However, the short-lived state is robustly identified, with a ca 20 to 30% occupancy and 40 to 70 ms dwell time, in all model sizes between 4 to 9 states (*SI Appendix*, Fig. S6), independent of expression system (cTF-Halo or p_w_TF-Halo in wt or cTF-Halo background, Dataset S1, Tabs 4, 12, and 13). Thus, this short-lived RNC-bound state is robustly distinct and independent from the others, and probably represents an average (50 ms) of a distribution of short TF-RNC binding events.

The average long-lived binding is 1 to 2 s, as determined independently both from the more complex models (≥4 states, *SI Appendix*, Fig. S6) in the 5 ms tracking data, and from the coarse-grained models in the 60 ms tracking data (*SI Appendix*, Fig. S11). This is significantly shorter than most in vitro measurements studying the binding kinetics of TF to individual RNC species (2, 22, 23). It should be noted though, that we are limited to only measuring average bindings to the large pool of different RNC species in the cell, and we cannot exclude that there exist high-affinity bindings lasting for up to tens of seconds. However, if such long binding events exist, they are not frequent enough to be separated in the HMM fitting procedure and they have little influence on the average bound time. Instead, our results suggest that TF mostly displays a more transient binding and rebinding cycle, where it stays associated with RNCs only for a fraction of the translation cycle. We speculate that the average binding observed in our in vivo data is shorter than the binding events measured in many in vitro studies because of relatively rapid changes in binding affinities in vivo due to the continuously growing nascent peptide. In line with our findings, selective Ribo-Seq, which also reflects the actual in vivo activity of TF, indicates that binding and unbinding is a dynamic process, possibly with many TF binding events per translation event, as the coverage of TF on transcripts is not uniform (8). Since our data support a model in which TF and SRP competes for RNC binding, we propose that these transient binding dynamics better explain the role of TF as a promiscuous chaperone with a large substrate pool overlapping with other processing factors (13, 44). If TF would stay bound to the majority of RNCs for up to tens of seconds (1, 23, 45), this would in practice yield most RNCs inaccessible to other essential processing factors, which would be detrimental to the cell. Promiscuous, but dynamic, binding to practically all potential targets is likely a general feature of biological systems, but has been difficult to study with conventional binding assays since it requires in situ studies at very high time resolution.

Although HMM fitting of diffusion trajectory data enables separation of states not only based on diffusion rates, but also on differences in transition frequencies, this work showcases this in a biological context. This was achieved thanks to the broad range of binding affinities exerted by TF to different RNC species in the cell, but also, due to the high temporal resolution of our data. Already in 2016, Yang et al. performed SPT of TF labeled with the photoconvertible fluorescent protein mEos3.2, using 60 ms camera exposure times. Yang et al. identified a 3-state diffusion model of TF, with similar diffusion rates and occupancies as the model established in our work (D_1_ = 0.02 ± 0.01 µm^2^s^−1^ and Occ_1_ = 44 ± 1%; D_2_ = 0.18 ± 0.04 µm^2^s^−1^ and Occ_2_ = 36 ± 3%; D_3_ = 3.85 ± 0.14 µm^2^s^−1^ and Occ_3_ = 20 ± 2%. However, Yang et al. assigned state 1 to RNC binding (representing an average of all types of RNC binding affinities), and state 2 to a combination of TF binding to free 50S subunits and slightly faster diffusion of TF binding to free client proteins in the cytosol. Given our tracking results at 5 ms, and the finding that TF binds nontranslating ribosomes less efficiently than elongating ribosomes, we argue that their lower temporal resolution leads to a misinterpretation of state 2. Consequently, we propose that their assignment of only 44% RNC binding is an underestimation, and rather, that both states 1 and 2 comprise RNC bindings, where state 2 includes the shorter RNC bindings. This would sum up to 80% RNC binding, in good agreement with our results. In line with this reasoning, we do acknowledge that also our results are limited by the time resolution of data acquirement, and, hence, that a free-state dwell time of 40 ms and 27% occupancy might actually be an overestimation. More details on this are provided in *SI Appendix*, Note S2.

TF dimerizes with an apparent K_D_ between 1 to 18 µM and a half-life of 1 s in vitro, but it binds to ribosomes as a monomer (2, 46). Assuming that the cellular concentration is at least 40 µM (39, 41, 42) and with a free state occupancy of 27% (Fig. 5*A* and Dataset S1, Tab S13), we thus expect the concentration of free TF to be around 11 µM in our experiment, i.e., high enough for dimerization to occur at a significant level in vivo (46, 47). Strikingly, however, our SPT data show that the average time between RNC-binding events is only tens of milliseconds. Hence, with a half-life of 1 s for the dimer, we deem it unlikely that TF dimerizes between RNC-binding cycles in vivo. This finding further highlights the significance of single-particle experiments in exploring the dynamic nature of biomolecules.

With this work, we have provided an experimental scheme to study cotranslational processing of nascent chains in its native environment. We have directly measured longer and shorter RNC bindings of TF-Halo, and by genetic perturbations, measured the RNC-binding competition between SRP and TF. We find that the average TF-RNC binding in vivo is shorter than what was obtained in many in vitro measurements, studying the binding kinetics to individual RNC species (1, 45). Although we are limited to only providing an average binding time, and surely there could be specific RNCs with which TF stays associated for longer, we argue that, given the vast range of possible binding partners in the cell, all yielding different residence times, this average binding time may in fact be more insightful for the in vivo function of TF, than the residence time on individual target RNCs in vitro. This method can further be used for measuring the binding kinetics of other cotranslational processing factors to RNCs, and their dynamic interplay, in living *E. coli* cells.

## Methods

### Cloning and Strain Construction.

Cloning procedures involving PCR were performed using Q5 High-fidelity polymerase (New England Biolabs) according to the manufacturer’s protocol. For construction of the chromosomal TF-Halo fusion in *E. coli* K12 MG1655 (accession #U00096), the gene encoding HaloTag was inserted on the chromosome directly upstream of the stop codon of the *tig* gene, encoding TF, creating a C-terminal TF-Halo fusion (MG1655tig::halotagKan). The *halotag* gene was inserted along with a kanamycin resistance marker using lambda red recombineering (48) with pKD4_halotag (29) as template and primers tig_HaloIns_F and tig_del_R (*SI Appendix*, Table S14). The tig_HaloIns_F primer was designed such that the start codon of *halotag* is exchanged to GGC, creating a 1-glycine linker between TF and HaloTag.

For experiments with titration of TF-Halo levels, the fusion was expressed from a plasmid (pQE30lacIq_tig_halotag) under the T5 promoter with two lac operators, yielding IPTG-inducible expression. The plasmid was inserted in a strain lacking chromosomal TF (MG1655∆tig::kan). Similarly, the TF_FRK/AAA_-HaloTag mutant was expressed from the same vector (pQE30lacIq_tig_halotag_frk_aaa) in the TF knockout background. Construction of MG1655∆tig::kan and the two plasmids are described in ref. 28. For low constitutive expression of TF-Halo from a plasmid (p_w_TF-Halo), inverse PCR was performed on pQE30lacIq_tig_halotag to exchange the T5 promoter to the weak constitutive apFAB124 promoter and to switch to a weak Shine–Dalgarno sequence [TTCTCA instead of AGGAGG (29)] using primers pQE_SDw_F and p124_R (pQE30lacIq_p124_SDw_tig_halotag). The linear PCR product was phosphorylated with T4 Polynucleotide kinase and circularized using T4 DNA-ligase according to the manufacturer’s instructions (Thermo Scientific). All constructs were verified by Sanger Sequencing.

### Sample Preparation.

For SPT of chromosomally expressed TF-Halo, an overnight culture of MG1655tig::halotagKan was prepared from a glycerol stock in Luria Broth (LB) at 37 °C 200 rpm. The overnight culture was diluted 1:100 in fresh LB followed by incubation at 37 °C 200 rpm until OD_600_ reached 0.4 to 0.8. Cells were harvested and resuspended in EZ Rich Defined Medium (RDM) with 0.2% glucose (Teknova) and 0.2 µM JFX549 (a gift from Luke Lavis lab). Cells were labeled at 25 °C for 30 min, followed by washing with M9 medium with 0.2% glucose, incubation in RDM + glucose at 37 °C 200 rpm for 60 min, and three subsequent washing steps in M9 + glucose to remove any unbound JFX549. The culture was diluted to approximately OD_600_ 0.003 and spread onto a pad with 2% agarose (SeaPlaque GTG Agarose, Lonza) in RDM + glucose on a microscopy slide. The sample was put in the microscope surrounded by an incubator set to 37 ± 2 °C, and left for incubation for at least 100 min and a maximum of 200 min before imaging. For SPT of TF-Halo in Ksg-treated cells, the sample was incubated for 100 min after which 2 mg/ml Ksg was injected to the sample. Data were collected after 50 to 80 min Ksg treatment. For SPT in Pm-treated cells, 0.2 mg/ml Pm was injected to the sample after ca 120 min incubation, and data were collected after 80 to 110 min treatment. Pm treatment showed heterogeneous phenotypic effects (elongated cells and appearance of dark spots; see example in Fig. 7*E*). For stringency, any colonies without clear phenotypic effects were filtered out in the analysis.

Samples for SPT of L9-Halo [expressed chromosomally using MG1655rplI::halotagKan, constructed in reference (29)] were prepared similarly as the TF-Halo samples, but cells from a glycerol stock were streaked on Luria-Agar (LA) plates grown at 37 °C overnight and starting cultures were inoculated with ca 5 colonies instead of an overnight culture. The cells were labeled with 0.5 µM JFX549.

SPT of plasmid-expressed TF-Halo (pQE30lacIq_tig_halotag and pQE30lacIq_p124_SDw_tig_halotag) was performed in wt MG1655, MG1655tig::halotagKan, or MG1655∆tig::kan strains as implied. Sample preparation was performed as described above, with all growth media supplemented with 100 µg/ml ampicillin for maintenance of the plasmid. For induction of TF-Halo expression, IPTG was added to the RDM-agarose mixture during preparation of the agarose pad to a final concentration of either 25, 50, or 100 µM. Sample preparation for tracking the TF_FRK/AAA_-Halo mutant in MG1655∆tig::kan was performed similarly, without IPTG induction, yielding only leaky expression of TF_FRK/AAA_-Halo. Similarly, tracking of LacY-Halo was performed in MG1655 supplied with pQE30lacIq_lacY_halotag (lab collection) at leaky expression levels.

For tracking of SRP, LD655-labeled 4.5S RNA was electroporated into MG1655∆tig::kan carrying either pQE30lacIq_tig_halotag for IPTG-inducible expression of TF-Halo, or pQE30lacIq_halotag (lab collection) for inducible expression of only HaloTag. Preparation of LD655-4.5S RNA, electrocompetent cells, and electroporation was executed as described in ref. 31 and IPTG induction on the agarose pad was achieved as described above.

### Optical Setup and Imaging Conditions.

For widefield epifluorescence imaging, an inverted Ti2-E microscope (Nikon) with a CFI Plan Apo lambda 1.45/100× objective (Nikon) was used. The microscope was built into an H201-ENCLOSURE incubator hood H201-T-Unit-BL and controller (OKOlab), allowing imaging at 37±2 °C. Brightfield and fluorescence images were acquired with an iXon 897 Ultra EMCCD camera (Andor) with an additional 2× lens in front of it (DD20NLT, Diagnostic Instruments). Phase contrast images were acquired with the same iXon camera or with an Infinity 2-5 M camera (Lumenera). For imaging of HaloTag-fused proteins labeled with JFX549, either a 553 nm laser (SLIM-553L, 150 mW, Oxxius) or a 546 nm laser (2RU-VFL-P-2000-546-B1R, 2000 mW, MPB Communications) was used with a power density of 3 kW/cm^2^ on the sample plane and stroboscopic illumination with 3 ms laser pulses per 5 (TF-Halo and L9-Halo), 30 (LacY-Halo), or 60 ms (TF-Halo) camera exposure. JFX549-labeled TF-Halo was imaged at 5 and 60 ms. For TF-Halo imaging using camera exposures of 2.5 ms, 2.3 ms laser pulses were used. For tracking of LD655-labeled 4.5S RNA, a 639 nm laser (Genesis MX 639-1000 STM, Coherent) was used with a power density of 4.5 kW/cm^2^ on the sample plane and stroboscopic illumination with 1.5 ms laser exposure per 20 ms camera exposure. Between 500 to 1,000 fluorescence images were acquired per cell colony to create the fluorescence movies. To visualize SYTOX blue stain on cells electroporated with LD655-4.5S RNA, and thus identify and exclude any dead cells, they were imaged with a 405 nm laser (06-MLD 360mW, Cobolt) with a power density of 17 W/cm^2^ and continuous illumination with 21 ms camera exposure time.

The microscope was controlled using µManager and data acquisition was automated using custom-made µManager plugins. Between 30 to 90 positions on the agarose pad were recorded in one experiment, with each position containing minicolonies grown from single cells. Each tracking condition was repeated in at least three independent experiments, showing consistent results between replicates; see model outputs from each replicate and cumulated datasets for chromosomal TF-Halo expression in Dataset S1, Tabs 1–4.

### Data Analysis.

SPT data were analyzed using a custom MATLAB-based pipeline, as previously described (29, 31, 49). In short, segmentation masks of single cells were created based on phase contrast images using the Per Object Ellipse fit (POE) method for adaptive thresholding (50). Segmentation parameters were optimized based on which camera was used for phase-contrast imaging (iXon 897 or Infinity 2-5 M) to obtain equivalent segmentation performance. Each position was manually curated to remove any poorly segmented cells and cells that were placed outside the region of even fluorescence illumination within the field of view. For SRP tracking, any SYTOX-positive cells, i.e., cells that died during electroporation, were also removed. Segmentation masks were aligned with fluorescence images for tracking fluorophores on a per-cell basis, using the radial symmetry-based algorithm (51). Symmetric Gaussian PSF modeling and maximum a posteriori fitting was performed for dot localization refinement and for estimation of dot localization uncertainty to filter out any faulty dots (52). Dot detection and refinement parameters were optimized for each type of fluorophore (JFX549 and LD655) and laser used (546, 553, and 639 nm). Each detected dot was assigned to a segmented cell and once there was only one dot detected in a cell, the trajectory of said dot was built using the uTrack algorithm (53). Parameters were set to allow gaps in two consecutive frames (accounting for dots occasionally moving out of the focal plane), and a search radius of 20 pixels (1.6 µm), covering approximately the entire cell area.

To extract the diffusional behavior of particles and their kinetic parameters, an ensemble of trajectories with a minimal length of 5 steps (TF-Halo and L9-Halo) or 3 steps (SRP) were analyzed using a diffusion-based HMM algorithm, described in detail in Supplementary Note 11 in reference (52), and implemented as described in our previous work (29, 31). In short, an ensemble of trajectories was fitted to a predefined number of hidden states, corresponding to discrete states of diffusion with an average diffusion rate, steady state occupancy, and dwell time. The algorithm uses maximum-likelihood with 100 iterations and independent starting values for estimation of the diffusion rates and transition probabilities for any given model size. In addition, it makes explicit use of the dot localization uncertainties for localization refinement and accounts for motion blur, unlike other HMM-based algorithms, such as vbSPT (54). The data from each tracking experiment were analyzed separately as well as combined with biological replicates, yielding cumulated datasets containing at least 50 000 steps per experimental condition. The data were fitted to model sizes ranging from 2 to 9 diffusion states. 2-state models were obtained by coarse-graining all larger models (4 to 9 states) and calculating weighted averages of the fit parameters, described in closer detail in *SI Appendix*, Note S1. For TF-Halo, a threshold of 0.5 µm^2^s^−1^ was used for 2-state coarse-graining to separate free and ribosome-bound TF in all experimental conditions except for the Ksg and Pm treatments, where 0.8 µm^2^s^−1^ (Ksg) and 1 µm^2^s^−1^ (Pm) were chosen based on the diffusion of labeled 50S subunits under identical antibiotic treatment (Dataset S1, Tab 18 and ref. 29). For SRP, 0.8 µm^2^s^−1^ was used to separate RNC-bound from free SRP (4.5S RNA + Ffh) and free 4.5S RNA (in agreement with ref. 31). For L9-Halo in wt cells, a threshold of 0.5 µm^2^s^−1^ was used to separate translating and nontranslating ribosomes (50S subunits). For L9-Halo in Pm-treated cells, we used a threshold at 0.8 µm^2^s^−1^, based on the faster diffusion in the presence of Pm, to separate translating and nontranslating ribosomes.

## Supplementary Material

Appendix 01 (PDF)

Dataset S01 (XLSX)

Movie S1. Microscopy data of TF-Halo diffusion in live *E coli*. TF-Halo was expressed from the chromosome and labeled with JFX549. The movie (middle panel) was acquired with 5 ms camera exposure time and 3 ms illumination (546 nm) per image. For analysis, movies were aligned with cell outlines (segmented based on phase contrast images, bottom panel), and trajectories of single TF-Halo particles were built using the uTrack algorithm and HMM-fitted to a 2-state diffusion model with state 1 corresponding to slow diffusion and state 2 to fast diffusion (top panel). To reduce the risk of errors in the trajectory building, trajectories were recorded when there was only one fluorescent dot detected in a cell. Playback speed is 20 frames per second, i.e, 10 times slower than reality.

Movie S2. Microscopy data of TF_FRK/AAA_-Halo diffusion in live *E coli*. TFFRK/AAA-Halo was expressed from an IPTG-inducible plasmid at leaky expression level in a TF knockout strain and labeled with JFX549. The movie (middle panel) was acquired with 5 ms camera exposure time and 3 ms illumination (546 nm) per image. For analysis, movies were aligned with cell outlines (segmented based on phase contrast images, bottom panel), and trajectories of single TF-Halo particles were built using the uTrack algorithm and HMM-fitted to a 2-state diffusion model with state 1 corresponding to slow diffusion and state 2 to fast diffusion (top panel). To reduce the risk of errors in the trajectory building, trajectories were recorded when there was only one fluorescent dot detected in a cell. Playback speed is 20 frames per second, i.e, 10 times slower than reality.

Movie S3. Example of a TF-Halo trajectory HMM-fitted to a 4-state model displaying the RNC sampling behavior, i.e., frequent transitions between freely diffusing (state 3) and a slow-diffusing state (state 2). TF-Halo was expressed from the chromosome and labeled with JFX549. The movie was acquired with 5 ms camera exposure time and 3 ms illumination (546 nm) per image. State 1 corresponds to long RNC binding, state 2 to short RNC binding, and state 3 to free TF-Halo. Playback speed is 20 frames per second, i.e, 10 times slower than reality.

Movie S4. Example of a TF-Halo trajectory HMM-fitted to a 4-state model displaying long RNC binding. TF-Halo was expressed from the chromosome and labeled with JFX549. The movie was acquired with 5 ms camera exposure time and 3 ms illumination (546 nm) per image. State 1 corresponds to long RNC binding, state 2 to short RNC binding, and state 3 to free TF-Halo. Playback speed is 20 frames per second, i.e, 10 times slower than reality.

Movie S5. Example of a TF_FRK/AAA_-Halo trajectory HMM-fitted to a 4-state model. TF_FRK/AAA_-Halo was expressed from an IPTG-inducible plasmid at leaky expression level in a TF knockout strain and labeled with JFX549. The movie was acquired with 5 ms camera exposure time and 3 ms illumination (546 nm) per image. State 1 corresponds to long RNC binding, state 2 to short RNC binding, and state 3 to free TF-Halo. Playback speed is 20 frames per second, i.e, 10 times slower than reality.

## Data Availability

Microscopy images and videos data have been deposited in SciLifeLab Data Repository (https://doi.org/10.17044/scilifelab.27637464) (55).
