# Supplementary material for: Dynamic binding of the bacterial chaperone Trigger factor to translating ribosomes in Escherichia coli
Source: Proc Natl Acad Sci U S A. 2024 Dec 31;122(1):e2409536121. doi: 10.1073/pnas.2409536121 (PMC11725819; doi:10.1073/pnas.2409536121)
Supplement: Supplementary file 1 — Appendix 01 (PDF) [file pnas.2409536121.sapp.pdf]

Supplementary Information

Dynamic binding of the bacterial chaperone Trigger factor to translating ribosomes in *Escherichia coli*

Tora Hävermark, Mikhail Metelev, Erik Lundin, Ivan L. Volkov, Magnus Johansson
Department of Cell & Molecular Biology, Uppsala University
Correspondence: m.johansson@icm.uu.se

Table of contents

Fig. S1. Trajectory length distribution for chromosomally expressed TF-Halo tracked with 5 ms camera exposure time 3

Fig. S2. Akaike's information criterion (AIC) analysis of TF-Halo HMM-fitted models 4

Fig. S3. Example of erroneous connections in the uTrack trajectory building 5

Note S1: Calculation of coarse-grained and weighted mean model parameters 6

Table S1: 4-state model from Dataset S1, Tab 13 6

Table S2: 4-state model coarse grained to 2 states 6

Table S3: transition matrix for the 4-state model, providing the probability of a specific transition. The probabilities are rounded to three-digit precision. 6

Table S4: Pseudo count transition matrix for the 4-state model, rounded to natural numbers. 7

Table S5: Pseudo count transition matrix for the 4-state model, rounded to natural numbers. Elements that will be summed in the coarse graining are grouped. 7

Table S6: Pseudo count transition matrix for 4-state model coarse grained to 2 states, rounded to natural numbers 7

Table S7: The transition matrix (i.e., normalized pseudo count matrix) for the 4-state model coarse grained to 2 states, rounded to four digits. 7

Fig. S4. HMM models of TF-Halo and $TF_{FRK/AAA}$-Halo 9

Fig. S5. Levels of TF-Halo in strains used for SPT in Main Fig. 4 10

Fig. S6. Empirical assessment of HMMs of chromosomally expressed TF-Halo. 11

Fig. S7. HMM models of TF-Halo in different background strains. 12

Table S8: Number of trajectories containing transitions between states in low-level TF-Halo expression in a wt background data 13

Table S9: Total number of transitions between states in low-level TF-Halo expression in a wt background data 13

Table S10: Number of trajectories containing transitions between states in chromosomal TF-Halo data 13

Table S11: Total number of transitions between states in chromosomal TF-Halo data 13

Table S12: Number of trajectories containing transitions between states in $TF_{FRK/AAA}$-Halo data 13

Table S13: Total number of transitions between states in $TF_{FRK/AAA}$-Halo data 13

Fig. S8. State transitions in a subset of TF-Halo and $TF_{FRK/AAA}$-Halo trajectories fitted to 4-state models 14

Fig. S9. Fluxes of TF-Halo particles between states in HMM-fitted 4-state model.. 15

Fig. S10. HMM models of chromosomally expressed TF-Halo using different camera exposure times........... 16

Note S2: Evaluation of previous SPT of TF^{mE} (Yang *et al.* 2016) 17

Fig. S11. Convergence of dwell times in 2-state coarse-grained models.. 18

Fig. S12. HMM models of chromosomally expressed TF-Halo in cells treated with kasugamycin (Ksg) and puromycin (Pm).. 19

Note S3: Membrane-associated fraction of TF-Halo and L9-Halo with antibiotic treatment 20

Fig. S13. Membrane-associated fraction of TF-Halo and L9-Halo with antibiotic treatment........................20

Fig. S14. Experimentally derived radial profile of a membrane-bound protein (LacY-Halo)........................... 22

Table S14: List of primer sequences**.. 23**

Legend to Movie S1 24

Legend to Movie S2 24

Legend to Movie S3 24

Legend to Movie S4 24

Legend to Movie S5 24

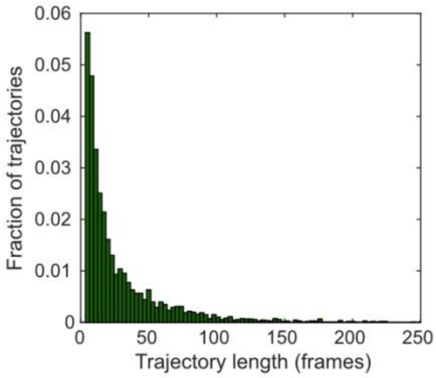

Fig. S1. Trajectory length distribution for chromosomally expressed TF-Halo tracked with 5 ms camera exposure time. Trajectories with at least 5 steps were included in the HMM fitting. n = 99,352 trajectory steps cumulated from 3 independent experiments. The mean trajectory length is 29 frames.

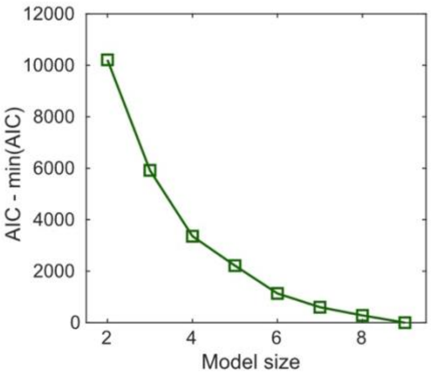

Fig. S2. Akaike's information criterion (AIC) analysis of TF-Halo HMM-fitted models. AIC scores, relative to the lowest one, for HMM fitting of trajectories of chromosomally expressed TF-Halo to different numbers of diffusion states (2-9). n = 99,352 trajectory steps cumulated from three independent experiments with 5 ms camera exposure time. The AIC score becomes smaller the more states are added.

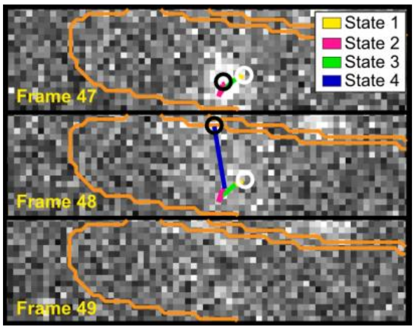

Fig. S3. Example of erroneous connections in the uTrack trajectory building. For trajectory building, the uTrack parameters have been set to only build trajectories in cells once there is only one fluorescent dot present in current and coming frames, to reduce the risk of linking two independent dots together. Occasionally, though, connections of two dots may occur, if for example a photobleaching event or a dot moving out of focus occurs concurrently as another dot appears elsewhere within the search radius in the next one or two frames. In frame 47 in the provided example, one dot is in the focal plane. In frame 48, it has moved out of focus, while another dot is appearing by the membrane, also out of focus, but anyway detected. In frame 49, none of the dots were detected. These erroneous connections contribute to the artefact fast diffusion state.

Note S1: Calculation of coarse-grained and weighted mean model parameters

Coarse graining is used to merge states in a multi-state model together to a less complex model. The states to be merged are determined by diffusion coefficient thresholds, where all discrete states between two user-defined thresholds will be merged. As an example, we look at the 4-state model for tracking of low-level plasmid expression of TF-Halo in wt MG1655 (*SI Appendix,* Table S1, also *Dataset S1,* Tab 13).

Table S1: 4-state model from Dataset S1, Tab 13

	State 1	State 2	State 3	State 4
**D ($\mu m^2 s^{-1}$)**	0.079 ± 0.002	0.214 ± 0.003	3.027 ± 0.029	11.793 ± 0.656
**Occupancy**	0.478 ± 0.012	0.274 ± 0.007	0.228 ± 0.006	0.020 ± 0.002
**Dwell time (s)**	0.813 ± 0.056	0.054 ± 0.001	0.042 ± 0.001	0.050 ± 0.005

Using a threshold at 0.5 $\mu m^2 s^{-1}$, we coarse grain the 4-state model to 2 states, where states 1 and 2 are merged into one state with D < 0.5 $\mu m^2 s^{-1}$, and states 3 and 4 are merged to one state with D > 0.5 $\mu m^2 s^{-1}$ (*SI Appendix,* Table S2).

Table S2: 4-state model coarse grained to 2 states

	State 1	State 2
**D ($\mu m^2 s^{-1}$)**	0.128 ± 0.002	3.724 ± 0.070
**Occupancy**	0.752 ± 0.006	0.248 ± 0.006
**Dwell time (s)**	0.140 ± 0.005	0.043 ± 0.001

The diffusion coefficients in the coarse-grained model are calculated as a sum of the merged states' diffusion coefficients, weighted by their occupancy. For example, the diffusion coefficient of state 1 in the coarse-grained model is calculated accordingly:

$$D_{CG,State\ 1} = \frac{0.079 \times 0.478 + 0.214 \times 0.274}{0.478 + 0.274} = 0.1282 \qquad (1)$$

Further, the occupancy of a coarse-grained state is simply given by the sum of the merged states in the multistate model. The dwell time (DT) of a state is calculated accordingly:

$$DT_{State} = \frac{\Delta t}{P_{exit}} \qquad (2)$$

where Δt is the exposure time in s and P_{exit} is the probability to exit the state, obtained either as the sum of all probabilities to exit the state, or conversely, as $1\text{-}P_{stay}$, where P_{stay} is the diagonal element for the state in the transition matrix. The transition matrix for the 4-state model is given in *SI Appendix,* Table S3.

Table S3: transition matrix for the 4-state model, providing the probability of a specific transition. The probabilities are rounded to three-digit precision.

	To state 1	To state 2	To state 3	To state 4
**From state 1**	0.994	0.001	0.005	0.000
**From state 2**	0.002	0.908	0.086	0.004
**From state 3**	0.006	0.113	0.880	0.001
**From state 4**	0.011	0.070	0.019	0.901

For the given dataset, the exposure time is 5 ms per frame. Thus, the state 1 dwell time in the 4-state model is given by:

$$DT_{State\ 1} = \frac{0.005\ s}{1 - 0.99385} = 0.813\ s \qquad (3)$$

Similarly, coarse-grained dwell times are calculated from the coarse-grained transition matrix. To obtain the coarse-grained transition matrix, we use a pseudo count transition matrix (obtained in the fitting procedure) of the 4-state model (*SI Appendix,* Table S4). The coarse-grained pseudo count transition matrix is obtained by summing the pseudo counts in the merged states, as illustrated in *SI Appendix,* Table S5. The coarse-grained pseudo count transition matrix is shown in *SI Appendix,* Table S6. Next, the matrix is normalized such that the sum of a row is 1 (*SI Appendix,* Table S7).

Table S4: Pseudo count transition matrix for the 4-state model, rounded to natural numbers.

	**To state 1**	**To state 2**	**To state 3**	**To state 4**
**From state 1**	45866	52	210	22
**From state 2**	64	23912	2264	99
**From state 3**	143	2474	19334	25
**From state 4**	20	130	35	1672

Table S5: Pseudo count transition matrix for the 4-state model, rounded to natural numbers. Elements that will be summed in the coarse graining are grouped.

	**To state 1**		**To state 2**	
**From state 1**	45866	52	210	22
	64	23912	2264	99
**From state 2**	143	2474	19334	25
	20	130	35	1672

Table S6: Pseudo count transition matrix for 4-state model coarse grained to 2 states, rounded to natural numbers

	**To state 1**	**To state 2**
**From state 1**	69893	2595
**From state 2**	2767	21065

Table S7: The transition matrix (i.e., normalized pseudo count matrix) for the 4-state model coarse grained to 2 states, rounded to four digits.

	**To state 1**	**To state 2**
**From state 1**	0.9642	0.0358
**From state 2**	0.1161	0.8839

Finally, the coarse-grained dwell times are calculated using Eq. 2:

$$DT_{CG,state\ 1} = \frac{0.005\ s}{1 - 0.9642} = 0.1397\ s \qquad (4)$$

$$DT_{CG,state\ 2} = \frac{0.005\ s}{1 - 0.8839} = 0.0431\ s \qquad (5)$$

The model parameter errors are obtained by bootstrapping the datasets with 300 iterations. The bootstrap error of a parameter is calculated as the standard deviation of the parameter in the bootstrap datasets. Similarly, the coarse-grained bootstrap error is calculated from bootstrapped coarse-grained models. After coarse-graining, we construct the final 2-state models by calculating the weighted mean of the coarse-grained multi-state models. We include model sizes of 4 to 9 states. The state parameters in the coarse-grained weighted mean model is calculated as a weighted mean of the parameter in all coarse-grained multistate models, where the weight (w) is given by:

$$w = \frac{1}{{E_b}^2} \qquad (6)$$

Where E_b is the bootstrap error. The standard deviation of the weighted average parameter is calculated with the same weights using the Matlab function *std*.

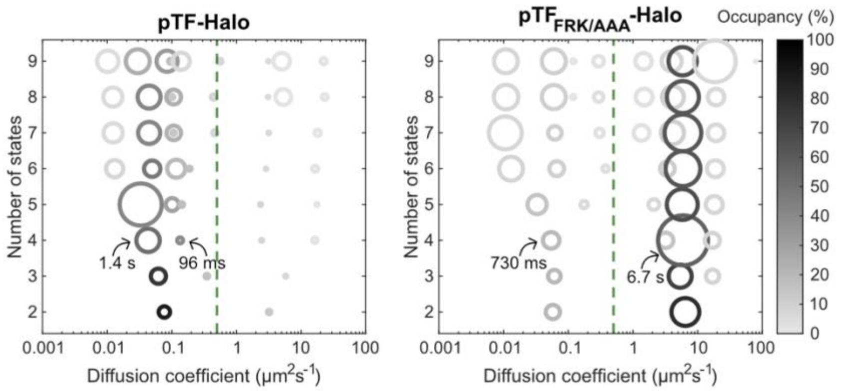

Fig. S4. HMM models of TF-Halo and TF$_{FRK/AAA}$-Halo. Leaky expression of TF-Halo and TF$_{FRK/AAA}$-Halo from plasmids in TF knockout cells. Circles are color-coded according to state occupancy and the area is proportional to the dwell time. Green lines mark a diffusion threshold of 0.5 μm^{2}s^{-1}, separating free and RNC-bound TF-Halo. n = 83,546 and 75,585 trajectory steps from 3 and 5 independent experiments, respectively. Full model outputs are shown in *Dataset S1,* Tabs 6-7.

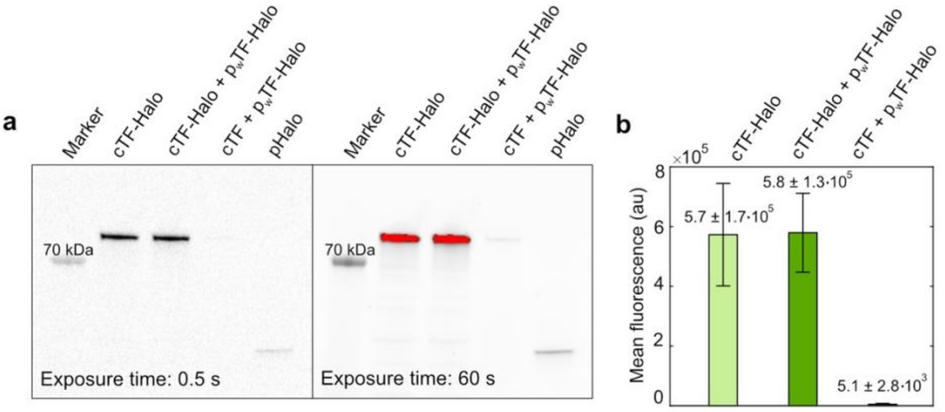

Fig. S5. Levels of TF-Halo in strains used for SPT in Main Fig. 4. a Example of SDS-PAGE gel imaged with green light to visualize fluorescence from TF-Halo labeled with JFX549. Levels of TF-Halo in each strain (cTF-Halo, cTF-Halo + p_wTF-Halo, cTF + p_wTF-Halo) were assessed by SDS-PAGE analysis of JFX549-labeled lysates. Strains were inoculated from glycerol stocks in Luria Broth (LB) and appropriate antibiotics and grown overnight at 37°C, 200 rpm. Overnight cultures were diluted 1:100 in LB and appropriate antibiotics and grown to $OD_{600} \approx 1.0$. 2 ml cell culture per 1 OD_{600} was pelleted and resuspended in 100 µl Bacterial Protein Extraction Reagent (B-PER, Thermo Scientific), supplemented with cOmplete Mini EDTA-free protease inhibitors (Sigma Aldrich) and 0.3 µM JFX549, followed by incubation at room temperature for 10 min. Lysates were mixed 1:1 with 2x Laemmli Buffer (Bio-Rad) and B-mercaptoethanol. SDS-PAGE was run on 4-20% Mini-PROTEAN TGX pre-cast gels at 180V for 45 min. Gels were imaged with ChemiDoc MP (Bio-Rad) using green illumination for visualization of JFX549 signal. At 0.5 s exposure (left), there was strong signal for chromosomally expressed TF-Halo and only a weak signal from weak plasmid expression in wt background (cTF + p_wTF-Halo). For better visualization of the weak signal, an additional image was acquired with 60 ms exposure time (right), at which the stronger signals became saturated (red pixels). Hence, the 0.5 s exposure images were used to quantify the fluorescence signal intensity. **b** Mean fluorescence intensities from TF-Halo in each strain. Intensities were measured by densitometry from images with 0.5 s camera exposure time. Chromosomal expression of TF-Halo yields approximately 100-fold more TF-Halo than weak expression from the p_wTF-Halo plasmid. Mean intensities were calculated from n = 5 independent experiments and error bars show the standard deviation. Intensities from individual experiments are shown in *Dataset S1,* Tab 27.

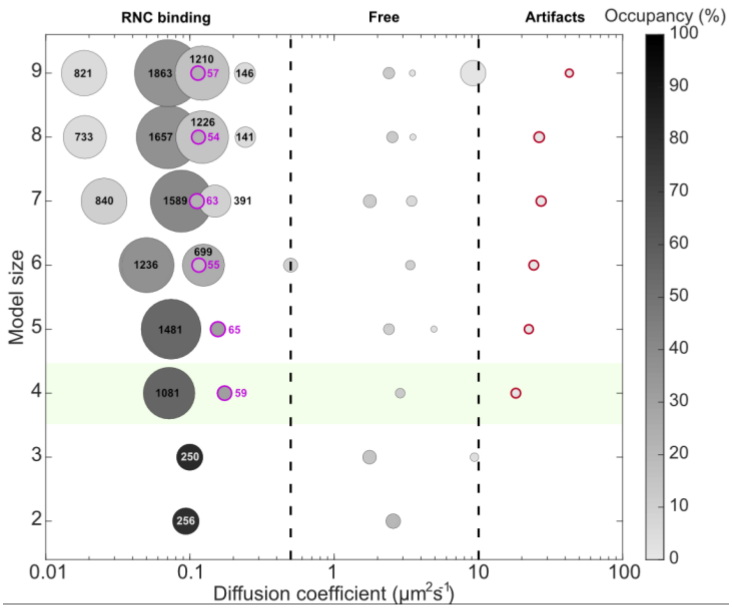

Fig. S6. Empirical assessment of HMMs of chromosomally expressed TF-Halo. HMM fitting of trajectories to different model sizes (2-9). The plot displays the same data as in Main Fig. 2, but with some highlighted features to better illustrate the assessment of the robustness of the models. Each model is resolved along the y-axis with discrete diffusion states represented by circles resolved along the x-axis based on diffusion rate. The 4-state model is highlighted in green. Circles are color-coded according to state occupancy and the area is proportional to the state dwell time. For the RNC-bound states, the dwell times are explicitly indicated. The 2- and 3-state models are significantly different from the more complex models with respect to the average state dwell time in the slow diffusion states. There, the slow state dwell time is approximately 200 ms, similar to the global average obtained from 2-state coarse graining of more complex models. Only in models with 4 states or more, we start observing common features. Dashed lines mark diffusion thresholds of 0.5 and 10 $\mu m^2 s^{-1}$, separating the three clusters of diffusion states (RNC binding, free TF-Halo and tracking artefacts), which appear in all models with ≥ 4 states. Circles outlined in red are the tracking artefact states and circles outlined in pink are the short-lived RNC-bound state. The short-lived RNC-bound state is apparent with similar dwell time and state occupancy in all models with ≥ 4 states, but not in the less complex models. With increased complexity, the long-lived slow state is merely separated into several states with similar dwell times (roughly 1 s). n = 99,352 trajectory steps cumulated from 3 independent experiments. Full HMM output is found in *Dataset S1*, Tab 4.

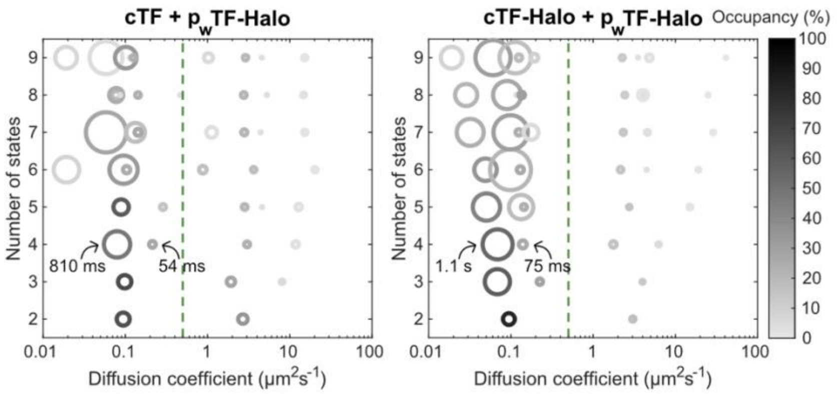

Fig. S7. HMM models of TF-Halo in different background strains. HMM fitting of 2-9 states of TF-Halo in the chromosomally tagged strain with additional weak plasmid expression (cTF-Halo + p_wTF-Halo) and in a wt strain with additional weak plasmid expression (cTF + p_wTF-Halo). n = 92,876 and 96,321 trajectory steps cumulated from 3 and 4 independent experiments for cTF-Halo + p_wTF-Halo and cTF + p_wTF-Halo, respectively. Circles are color-coded according to state occupancy and the area is proportional to the dwell time. Green lines mark a diffusion threshold of 0.5 $\mu m^2 s^{-1}$, separating free and RNC-bound TF-Halo. Full model outputs are shown in *Dataset S1,* Tabs 12-13.

Table S8: Number of trajectories containing transitions between states in low-level TF-Halo expression in a wt background data

Total # trj: 3464	To state 1	To state 2	To state 3	To state 4
**From state 1**	1392 (40.2%)*	37 (1.1%)**	199 (5.7%)	19 (0.5%)
**From state 2**	41 (1.2%)	179 (5.2%)	1126 (32.5%)	78 (2.3%)
**From state 3**	145 (4.2%)	1241 (35.8%)	220 (6.4%)	17 (0.5%)
**From state 4**	17 (0.5%)	103 (3.0 %)	23 (0.7%)	67 (1.9%)

* Diagonal elements are trajectories which do not contain any transitions.
** The fraction of the total number of trajectories is given in parentheses.

Table S9: Total number of transitions between states in low-level TF-Halo expression in a wt background data

	To state 1	To state 2	To state 3	To state 4
**From state 1**	0	37 (1.00)*	207 (1.04)	19 (1.00)
**From state 2**	42 (1.02)	0	2238 (1.99)	84 (1.08)
**From state 3**	148 (1.02)	2441 (1.97)	0	17 (1.00)
**From state 4**	17 (1.00)	107 (1.04)	25 (1.09)	0

* The ratio between number of transitions to number of trajectories displaying transitions between two states is given in parentheses.

Table S10: Number of trajectories containing transitions between states in chromosomal TF-Halo data

Total # trj: 3531	To state 1	To state 2	To state 3	To state 4
**From state 1**	1692 (47.9%)	34 (1.0%)	180 (5.1%)	7 (0.2%)
**From state 2**	74 (2.1%)	222 (6.3%)	1075 (30.4%)	32 (0.9%)
**From state 3**	100 (2.8%)	1178 (33.4%)	78 (2.2%)	91 (2.6%)
**From state 4**	31 (0.9%)	114 (3.2%)	34 (1.0%)	25 (0.7%)

Table S11: Total number of transitions between states in chromosomal TF-Halo data

	To state 1	To state 2	To state 3	To state 4
**From state 1**	0	36 (1.06)	185 (1.03)	7 (1.00)
**From state 2**	77 (1.04)	0	2448 (2.28)	33 (1.03)
**From state 3**	103 (1.03)	2598 (2.21)	0	101 (1.11)
**From state 4**	31 (1.00)	122 (1.07)	34 (1.00)	0

Table S12: Number of trajectories containing transitions between states in $TF_{FRK/AAA}$-Halo data

Total # trj: 4175	To state 1	To state 2	To state 3	To state 4
**From state 1**	557 (13.3%)	62 (1.5%)	23 (0.6%)	6 (0.1%)
**From state 2**	78 (1.9%)	477 (11.4%)	2 (0.0%)	0 (0%)
**From state 3**	0 (0%)	18 (0.4%)	2543 (60.9%)	0 (0%)
**From state 4**	18 (0.4%)	2 (0.0%)	22 (0.5%)	426 (10.2%)

Table S13: Total number of transitions between states in $TF_{FRK/AAA}$-Halo data

	To state 1	To state 2	To state 3	To state 4
**From state 1**	0	71 (1.15)	23 (1.00)	6 (1.00)
**From state 2**	89 (1.14)	0	2 (1.00)	0 (0)
**From state 3**	0 (0)	18 (1.00)	0	0 (0)
**From state 4**	18 (1.00)	2 (1.00)	22 (1.00)	0

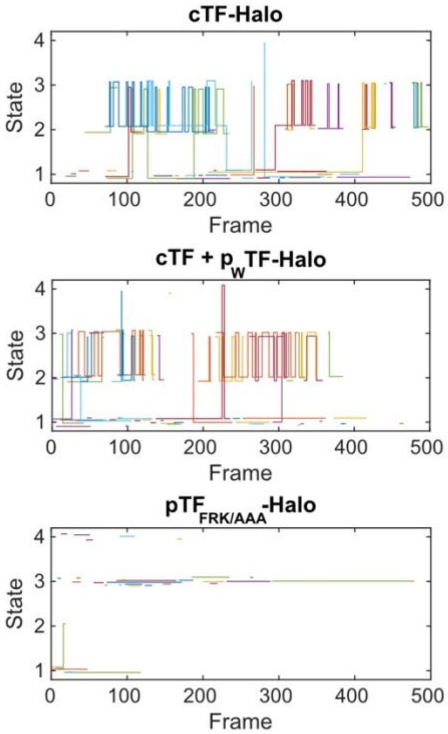

Fig. S8. State transitions in a subset of TF-Halo and $TF_{FRK/AAA}$-Halo trajectories fitted to 4-state models. a Trajectories detected in cells from one SPT movie of chromosomally expressed TF-Halo (cTF-Halo), from low TF-Halo expression in the wt TF background (cTF + p_wTF-Halo) and from the FRK/AAA mutant. For TF-Halo, the most frequent transitions are between states 3 and 2, i.e., the free and short-lived RNC-bound state. State 1 is longer RNC bindings. The FRK/AAA mutant, compromised in ribosome binding, does not exhibit fast transitions between fast and slow diffusion.

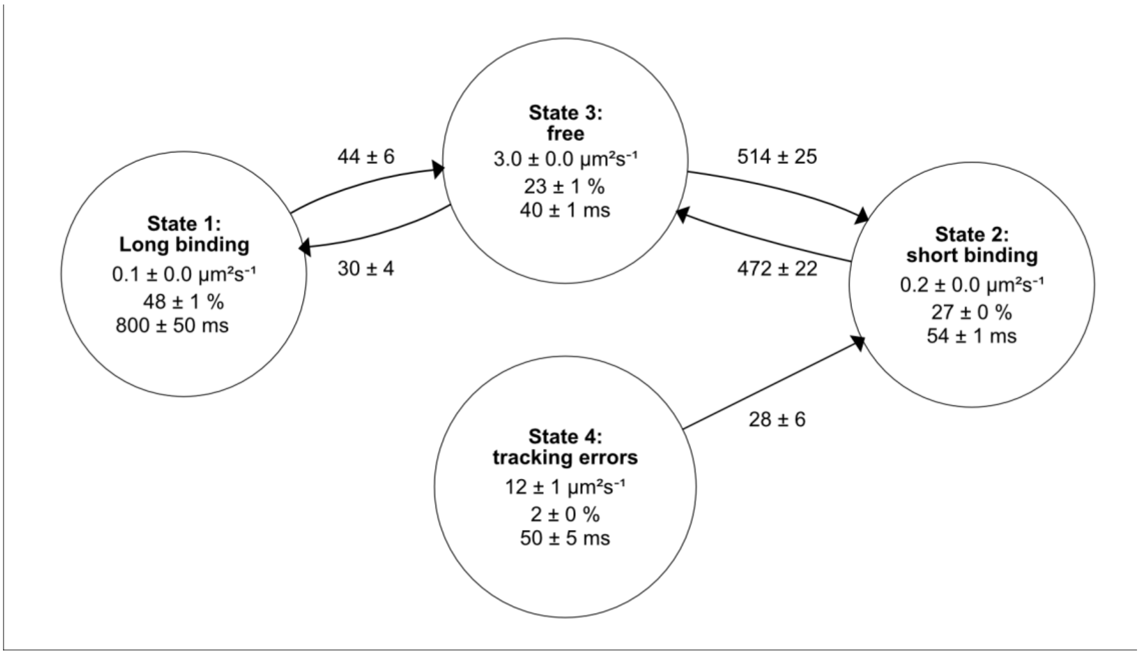

Fig. S9. Fluxes of TF-Halo particles between states in HMM-fitted 4-state model. Direction of fluxes are implied by the arrows. Fluxes are given as the percentage of the total population of TF particles transitioning between two states per second ($\%s^{-1}$). Transitions between the free and the short-lived RNC-bound state is predominant. Fluxes between states with less than 25 $\%s^{-1}$ were excluded from the chart. As such, although transitions between state 1 and 2 (long- and short-lived RNC bindings) occur, they are less frequent ($<25\%s^{-1}$) than transitions from state 3 to each respective state. This could be an inherent feature of the RNC binding of TF. However, we cannot exclude that transitions between states 1 and 2 are underrepresented as it is harder to distinguish states with similar diffusion coefficients in the HMM fitting. Data is from low-level plasmid expression of TF-Halo in a wt background tracked at 5 ms, n = 96,321 trajectory steps cumulated from 4 independent experiments. Errors for diffusion coefficients, occupancies and dwell times are bootstrap-estimated standard errors. For fluxes, the errors are propagated from the state occupancy and transition frequency bootstrap errors.

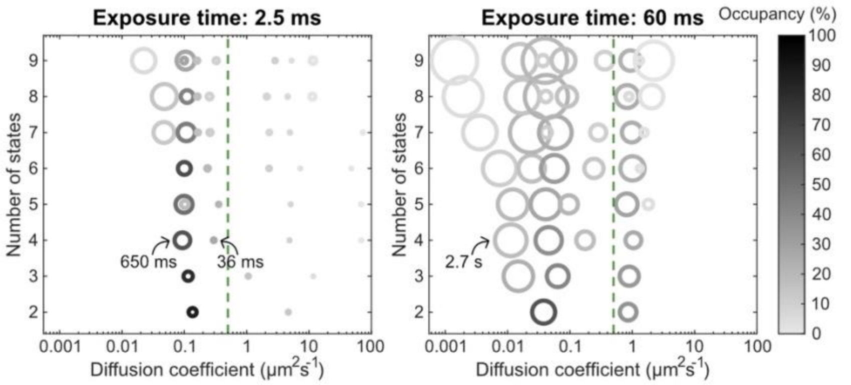

Fig. S10. HMM models of chromosomally expressed TF-Halo using different camera exposure times. With 2.5 ms exposure time, long and short RNC-bound states are resolved, similarly to the 5 ms data (main Fig. 2a). With 60 ms, the exposure time equals the average short-lived RNC-bound time and is longer than the average time between binding events (40 ms), and consequently, the short RNC bindings cannot be distinguished in the HMM fitting. We note that for "free" TF-Halo with 60 ms exposures, the apparent diffusion becomes slower and dwell times become longer. This is attributed to averaging between the free and short-lived RNC bindings, as discussed in *SI Appendix,* Note S2. Circles are color-coded according to state occupancy and the area is proportional to the dwell time. Green lines mark a diffusion threshold of 0.5 $\mu m^2 s^{-1}$. n = 86,929 and 83,114 trajectory steps for 2.5 and 60 ms data, respectively, each cumulated from 3 independent experiments. For comparison, the x axes were set to [0.001, 100] for both the 2.5 and the 60 ms plot. However, model size 6 in 2.5 ms tracking contains a low occupancy (< 0.1%) slow artefact state with D = 10^{-6} $\mu m^2 s^{-1}$, not included in the plot. Full model outputs are shown in *Dataset S1,* Tabs 14-15.

Note S2: Evaluation of previous SPT of TF^{mE} (Yang *et al.* 2016)

While Yang *et al.* (2016) provided a comprehensive view on the *in vivo* function of TF, fused to the photoconvertible fluorescent protein mEos3.2 (TF^{mE}), and validated their SPT data using several approaches, we believe that recent progress in SPT data analysis and, importantly, protein labeling with bright and photostable organic fluorescent dyes, provide further insight.

First, we acknowledge that Yang *et al.* also identified a three-state model, with similar diffusion rates and occupancies as the model established in our work ($D_1 = 0.02 \pm 0.01$ µm^{2}s^{-1} and $Occ_1 = 44 \pm 1\%$; $D_2 = 0.18 \pm 0.04$ µm^{2}s^{-1} and $Occ_2 = 36 \pm 3\%$; $D_3 = 3.85 \pm 0.14$ µm^{2}s^{-1} and $Occ_3 = 20 \pm 2\%$; derived from PDF fitting of the displacement length distribution). However, we draw different conclusions regarding the biological interpretations of states 1 and 2. Yang et al. assigned state 1 to RNC binding (both target and non-target bindings), and state 2 to a combination of TF binding to free 50S subunits and slightly faster diffusion of TF binding to free client proteins in the cytosol. These conclusions were based on experiments showing that D_2 becomes faster when TF^{mE} is overexpressed (0.7 µm^{2}s^{-1}) and when the FRK/AAA mutant is tracked (ca 0.9 µm^{2}s^{-1}), arising from a larger contribution of faster-diffusing TF-client protein complexes compared to TF-50S interactions under these experimental conditions. Based on the data available at the time, these conclusions are reasonable. However, with data of higher temporal resolution at hand, we argue that these effects can be explained differently.

Using a camera exposure time of 5 ms, we find two different binding modes of TF-Halo to RNCs: longer bindings (ca 1 s, state 1) and short bindings (ca 50 ms, state 2). Importantly, the average dwell time in the free state is only 40 ms. Thus, with a time-lapse of 60 ms per frame, used by Yang et al., the transitions between RNC binding and the free state will most often be missed and instead result in an "averaged" diffusion state, with a diffusion rate faster than that of RNCs, but slower than free TF. Hence, we argue that state 2 in Yang et al., is such an "averaged" state of fast transitions between free and RNC-bound TF, whereas state 1 separates the longer RNC-bound events. To highlight this effect, we also performed SPT at 60 ms per frame. Indeed, also in our experiment, the free state is averaged out, resulting in an apparent diffusion of only ca 1 µm^{2}s^{-1} (*SI Appendix*, Fig. S10, *Dataset S1*, Tabs 5 and 15), and the discrepancy between longer and shorter RNC bindings is lost. Further, the diffusion rate of free $TF_{FRK/AAA}$-Halo, where the fast transitions between the freely diffusing state and RNC binding are absent, is ca 6 µm^{2}s^{-1}, compared to ca 3 µm^{2}s^{-1} for wt TF-Halo. This suggests that also at 5 ms per frame, there is some averaging due to transitions that are beyond our temporal resolution. It is therefore conceivable that our model also overestimates the dwell times in the short-lived RNC state and in the free state. Based on this, we argue that the estimation of only 44% RNC-bound TF^{mE} in Yang *et al.* is an underestimation, and that, according to our model, TF is RNC-bound at ca 75% in cells with near-native TF levels.

Fusing TF to HaloTag-JFX549, with superior brightness and photostability compared to mEos3.2, allowed us to use shorter camera exposure times, record longer trajectories, and consequently, capture a lot more transitions between diffusion states. Since the HMM-estimated dwell times are based on transition frequencies between the different diffusion states, having a dataset with more transitions yields more reliable dwell times. With that said, we find good agreement between the state 1 dwell-time estimates in both studies, with ca 800 ms in our work and the HMM-estimated 1.4 s in Yang et. al, obtained by applying vbSPT, further corroborating that state 1 in Yang *et al*. captures the longer RNC bindings. However, rather than reporting only the HMM-derived dwell time, Yang *et al.* highlighted an average dwell time of 0.2 s, which was obtained by fitting the distribution of residence times of all displacements below a threshold of 220 nm (i.e., all slow-diffusing events). Interestingly, they commented that this dwell time contains contributions of residence times from state 2, as the displacement distributions of states 1 and 2 are overlapping. With our reasoning, that their state 2 is a mixture of short RNC bindings and free TF diffusion, it is reasonable that this approach yields a similar dwell time to the one we obtain by coarse-graining our data into one RNC-bound state (130 ms).

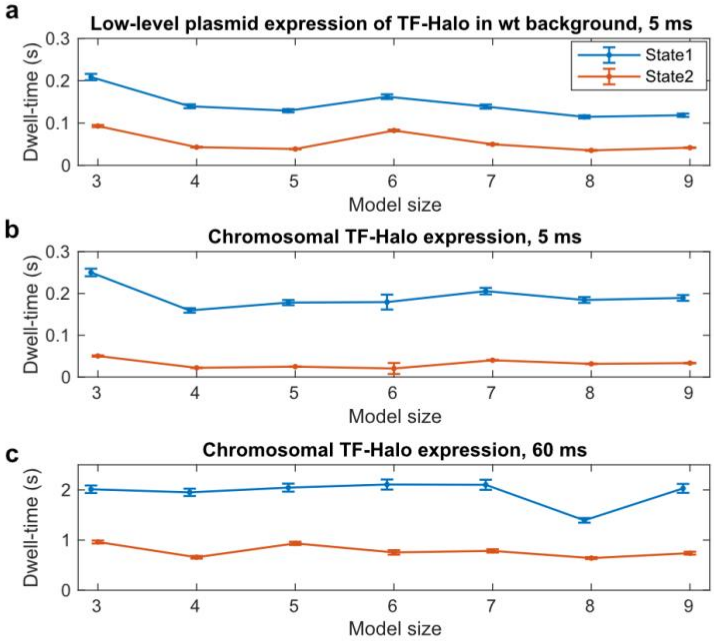

Fig. S11. Convergence of dwell times in 2-state coarse-grained models. a,b Coarse-grained models of TF-Halo tracked with 5 ms camera exposures, expressed at low levels from a plasmid in a wt background (**a**) or from the chromosome (**b**). **c** Coarse-grained models of TF-Halo tracked with 60 ms camera exposures. At 5 ms exposures, the coarse-grained average RNC-bound time is around 0.2 s, for all model sizes, using both expression systems, confirming that coarse graining yields robust dwell time estimates. State transitions will be picked up in the trajectory analysis only if the particle was tracked for at least a few frames before and after the transition, yielding an approximate temporal resolution of ca 4 frames, i.e., 20 ms for the 5 ms tracking, and ca 200 ms for the 60 ms tracking. Thus, in the 60 ms tracking, all binding events shorter than approximately 200 ms are filtered out from the average RNC-bound time in the 2-state coarse-grained model (also discussed in *SI Appendix*, Note S2). As the short-lived bindings (≤ 60 ms) are the most frequent type of interaction with the RNCs (ca 20 short bindings per 1 long binding event, *SI Appendix,* Fig. S9), they have a significant influence on the global average binding time obtained in the coarse-grained 2-state model in the 5 ms tracking data (**a** and **b**). Thus, by coarse-graining the 60 ms models into 2 states, the influence of the high-frequency short-lived RNC bindings disappears, yielding an average dwell time for the longer bindings alone. The average bound time in the 60 ms tracking data is around 2 s, independent of model size used for coarse-graining (**c**), confirming that these coarse-grained dwell times also are robust. With this, we are confident that the longer bindings last, on average, for 1-2 s, as determined from this coarse-graining analysis of the 60 ms data, and, as observed for individual states in more complex models in the 5 ms tracking data (*SI Appendix,* Fig. S6). n = 99,352; 96,321; 83,114 trajectory steps cumulated from 3, 4, and 3 independent experiments for 5 ms cTF-Halo, 5 ms cTF + p_wTF-Halo, and 60 ms cTF-Halo, respectively. Error bars were obtained by bootstrapping with 300 iterations. Full model outputs are shown in *Dataset S1,* Tabs 13, 4 and 15. Note that the coarse-grained models presented in the main text (and Dataset S1, Tab 5) were obtained by further calculating the weighted means of the coarse-grained parameters from all model sizes between 4-9 states (see details on the procedure in *SI Appendix*, Note S1).

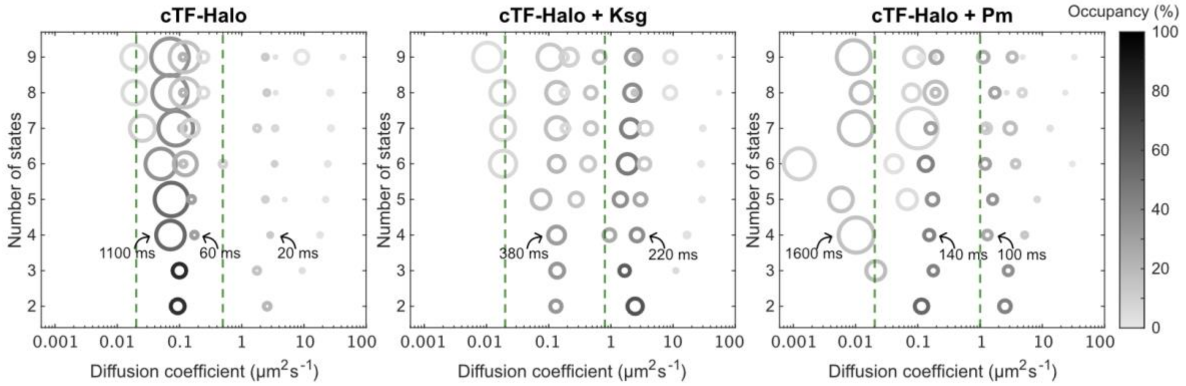

Fig. S12. HMM models of chromosomally expressed TF-Halo in cells treated with kasugamycin (Ksg) and puromycin (Pm). The cTF-Halo plot (left) is the same data as shown Main Fig. 2a and is displayed here as a reference for the effect of antibiotic treatment. Upon Ksg treatment, dwell times in the free states become longer and the slow states are less populated. A similar, but less pronounced effect is observed on the free state dwell time with Pm treatment. Additionally, in the presence of Pm, substantially populated diffusion states of very slow diffusion rates appear. Circles are color-coded according to state occupancy and the area is proportional to the dwell time. Green lines mark the diffusion thresholds used for 2- and 3-state coarse-graining. n = 99,352; 85,146 and 67,028 trajectory steps cumulated from 3, 3 and 5 independent experiments, for untreated, Ksg-treated and Pm-treated samples, respectively. Full model outputs are shown in *Dataset S1,* Tabs 4, 16, and 19.

Note S3: Membrane-associated fraction of TF-Halo and L9-Halo with antibiotic treatment

In the HMM fitting of TF-Halo tracking data from puromycin-treated cells, highly-populated (ca 20%) diffusion states with approximately 10-fold slower diffusion (ca 0.01 $\mu m^2 s^{-1}$) than the normal RNC-bound states appear (*SI Appendix,* Fig. S12). Such states also appear in more complex models (≥ 8 states, *SI Appendix,* Fig. S12) for TF-Halo in untreated cells, but with a lower occupancy. These slow-diffusing states are distributed along the cellular membrane (*SI Appendix,* Fig. S13a-e, below). In order to estimate the fraction of slow-diffusing membrane-proximate TF-Halo, we coarse-grained 9-state models with two thresholds, D_{thr1} = 0.02 $\mu m^2 s^{-1}$ and D_{thr2} = 0.5, 0.8 or 1 $\mu m^2 s^{-1}$ for TF-Halo tracking data in untreated cells, Ksg-treated and Pm-treated cells, respectively, thus generating a 3-state model that separates free TF-Halo ($D_3 > D_{thr2}$) from slow cytosolic TF-Halo ($D_{thr1} < D_2 > D_{thr2}$) and slow membrane-associated TF-Halo ($D_1 < D_{thr2}$). Although the 3-state models do not perfectly separate membrane-associated and cytosolic slow diffusion (by comparison of the experimental distributions with the membrane and cytosolic profiles), it is a coarse estimate of the membrane-associated fraction of TF-Halo (Fig. S12f). As such, we find that roughly 5% of TF-Halo is found close to the membrane in untreated cells and in Ksg-treated cells. In Pm-treated cells, however, there is a significant increase in the membrane-associated state (roughly 20%). In contrast, when inspecting the membrane-associated fraction of L9-Halo, there is no significant increase upon Pm treatment. Assuming that the cellular levels of TF and ribosomes are roughly equimolar or that TF is in excess over ribosomes, we conclude that the significant redistribution of TF-Halo towards the membrane upon Pm-treatment is independent of ribosomes.

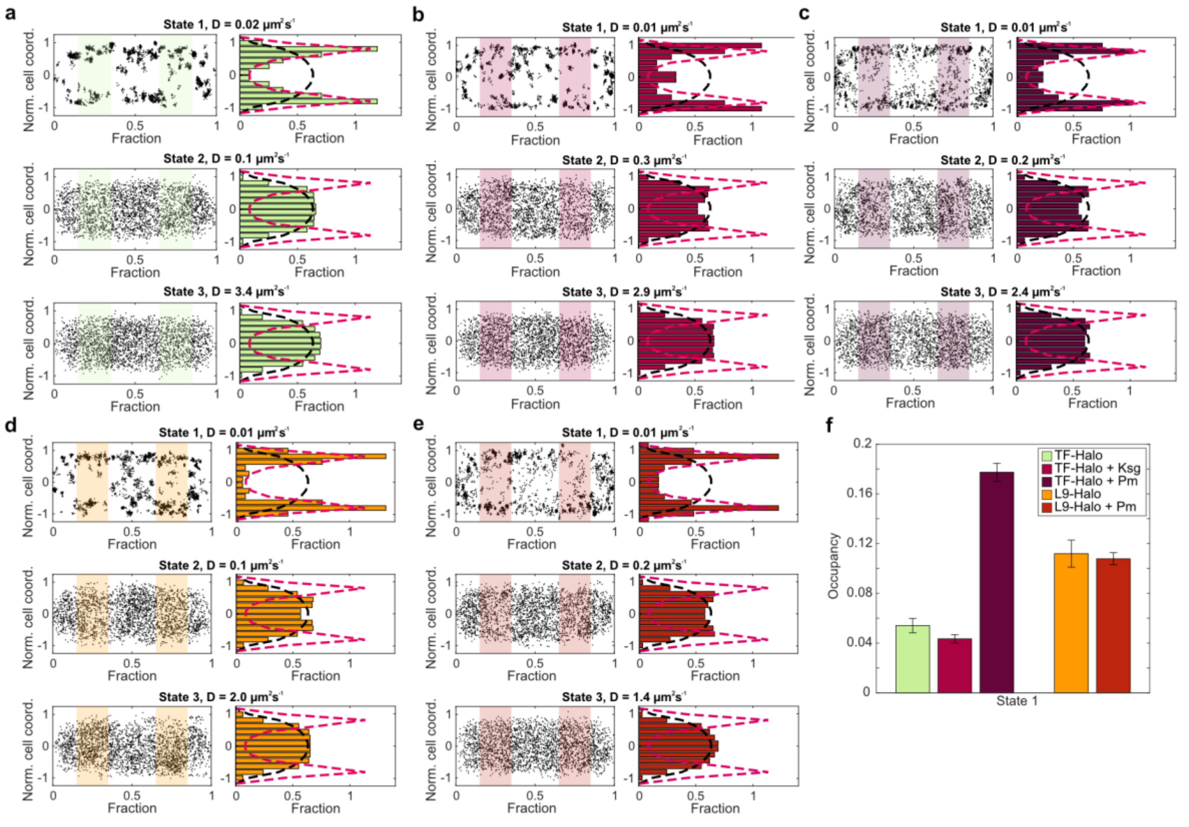

Fig. S13. Membrane-associated fraction of TF-Halo and L9-Halo with antibiotic treatment. a-e Radial distributions in 3-state models of TF-Halo without antibiotics (**a**), with Ksg (**b**) and with Pm (**c**) and L9-Halo without antibiotics (**d**) and with Pm (**e**). The left panels show coordinates of detected dots assigned to each state in a normalized cell geometry. 3,000 dots were selected at random for plotting, i.e., the plots do not reflect the relative state occupancies. The left panels show the radial distribution of all dots in the shaded

areas (to exclude the cell poles). The magenta dashed line is the radial profile of a membrane-bound protein derived experimentally from LacY-Halo tracking (*SI Appendix*, Fig. S14 and the black dashed line is a theoretically derived profile of a cytosolic particle. **f** Occupancy in the membrane-associated state (state 1) for all experimental conditions in **a-e**. Data is from 9-state models coarse-grained to 3 states with thresholds D_{thr1} = 0.02 and D_{thr2} = 0.5 (TF-Halo and L9-Halo without antibiotics), 0.8 (TF-Halo + Ksg and L9 + Pm) or 1 (TF-Halo + Pm) $\mu m^2 s^{-1}$. n = 99,352; 85,146; 67,028; 72,944; 90,956 trajectory steps cumulated from 3, 3, 5, 3, and 4 independent experiments, for untreated TF-Halo, TF-Halo + Ksg, TF-Halo + Pm, untreated L9-Halo, and L9-Halo + Pm, respectively. Full HMM output and 3-state coarse-grained models are found in Dataset S1, Tabs 4 and 16-S19. Error bars in **f** are bootstrap errors from the coarse-grained 9-state models.

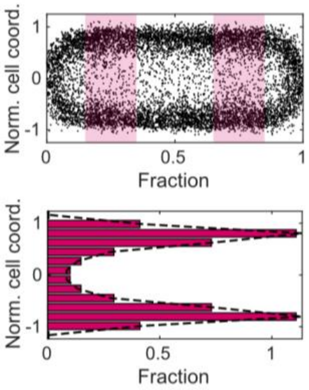

Fig. S14. Experimentally derived radial profile of a membrane-bound protein (LacY-Halo). SPT data of LacY-Halo was fitted to a 1-state HMM model to obtain the spatial distribution of a membrane-bound protein. The top panel shows coordinates of detected dots in a normalized cell geometry. 10,000 dots were selected at random for plotting. The bottom panel shows the radial distribution of all dots found in the shaded areas (to exclude the cell poles). The black dashed line is the membrane profile based on this LacY-Halo short-axis distribution. n = 72,760 steps cumulated from 4 independent experiments. Full HMM output is found in *Dataset S1*, Tab 26.

Table S14: List of primer sequences

Name	Sequence, 5’ -> 3’
tig_HaloIns_F	aaagaaaccactttcaacgagctgatgaaccagcaggcgggcgcagaaatcggtactggc
tig_del_R	acgggcctttgtgcgaatttagcgcgttatgctgcgtaaagtgtaggctggagctgcttc
pQE_SDw_F	tttcacacagaattcattaaagtttcttaattaactatgcaagtttcagttgaaac
p124_R	atggctgtaagtattcgccgcaagggataaatgtcgatttctcgaggtgaagacgaaagg

Legend to Movie S1

Microscopy data of TF-Halo diffusion in live *E coli*. TF-Halo was expressed from the chromosome and labeled with JFX549. The movie (middle panel) was acquired with 5 ms camera exposure time and 3 ms illumination (546 nm) per image. For analysis, movies were aligned with cell outlines (segmented based on phase contrast images, bottom panel), and trajectories of single TF-Halo particles were built using the uTrack algorithm and HMM-fitted to a 2-state diffusion model with state 1 corresponding to slow diffusion and state 2 to fast diffusion (top panel). To reduce the risk of errors in the trajectory building, trajectories were recorded when there was only one fluorescent dot detected in a cell. Playback speed is 20 frames per second, i.e, 10 times slower than reality.

Legend to Movie S2

Microscopy data of $TF_{FRK/AAA}$-Halo diffusion in live *E coli*. $TF_{FRK/AAA}$-Halo was expressed from an IPTG-inducible plasmid at leaky expression level in a TF knockout strain and labeled with JFX549. The movie (middle panel) was acquired with 5 ms camera exposure time and 3 ms illumination (546 nm) per image. For analysis, movies were aligned with cell outlines (segmented based on phase contrast images, bottom panel), and trajectories of single TF-Halo particles were built using the uTrack algorithm and HMM-fitted to a 2-state diffusion model with state 1 corresponding to slow diffusion and state 2 to fast diffusion (top panel). To reduce the risk of errors in the trajectory building, trajectories were recorded when there was only one fluorescent dot detected in a cell. Playback speed is 20 frames per second, i.e, 10 times slower than reality.

Legend to Movie S3

Example of a TF-Halo trajectory HMM-fitted to a 4-state model displaying the RNC sampling behavior, i.e., frequent transitions between freely diffusing (state 3) and a slow-diffusing state (state 2). TF-Halo was expressed from the chromosome and labeled with JFX549. The movie was acquired with 5 ms camera exposure time and 3 ms illumination (546 nm) per image. State 1 corresponds to long RNC binding, state 2 to short RNC binding, and state 3 to free TF-Halo. Playback speed is 20 frames per second, i.e, 10 times slower than reality.

Legend to Movie S4

Example of a TF-Halo trajectory HMM-fitted to a 4-state model displaying long RNC binding. TF-Halo was expressed from the chromosome and labeled with JFX549. The movie was acquired with 5 ms camera exposure time and 3 ms illumination (546 nm) per image. State 1 corresponds to long RNC binding, state 2 to short RNC binding, and state 3 to free TF-Halo. Playback speed is 20 frames per second, i.e, 10 times slower than reality.

Legend to Movie S5

Example of a $TF_{FRK/AAA}$-Halo trajectory HMM-fitted to a 4-state model. $TF_{FRK/AAA}$-Halo was expressed from an IPTG-inducible plasmid at leaky expression level in a TF knockout strain and labeled with JFX549. The movie was acquired with 5 ms camera exposure time and 3 ms illumination (546 nm) per image. State 1 corresponds to long RNC binding, state 2 to short RNC binding, and state 3 to free TF-Halo. Playback speed is 20 frames per second, i.e, 10 times slower than reality.
